# The Scalp Microbiome–Hair Axis: Mechanisms and Therapeutic Translation

**DOI:** 10.34133/research.1409

**Published:** 2026-08-17

**Authors:** Ting Li, Dongni Yao, Feng Cheng, Xiaozhou Mou, Qiong Bian

**Affiliations:** ^1^ Center for Rehabilitation Medicine, Rehabilitation & Sports Medicine Research Institute of Zhejiang Province, Department of Rehabilitation Medicine, Zhejiang Provincial People’s Hospital, Affiliated People’s Hospital, Hangzhou Medical College, Hangzhou, Zhejiang, China.; ^2^ College of Bioengineering, Zhejiang University of Technology, Hangzhou 310014, Zhejiang, China.; ^3^ National and Local Joint Engineering Research Center for Biomanufacturing of Chiral Chemicals, College of Biotechnology and Bioengineering, Zhejiang University of Technology, Hangzhou 310014, China.; ^4^ Key Laboratory of Bioorganic Synthesis of Zhejiang Province, College of Biotechnology and Bioengineering, Zhejiang University of Technology, Hangzhou 310014, China.; ^5^ Center for Plastic & Reconstructive Surgery, Department of Dermatology, Zhejiang Provincial People’s Hospital, Affiliated People’s Hospital, Hangzhou Medical College, Hangzhou 310014, Zhejiang, China.

## Abstract

Hair loss is common and can markedly impair quality of life, yet the contribution of the scalp microbiome to alopecia remains fragmented across disease subtypes and mechanistic levels. This review aims to construct a clinically relevant and mechanistically coherent framework for the scalp microbiome–hair axis. We first summarize the composition and physiological functions of the scalp microbiome, including its roles in barrier integrity, immune regulation, and nutrient metabolism. We then compare dysbiosis patterns across androgenetic alopecia, alopecia areata, seborrheic dermatitis-associated hair loss, and folliculitis/scarring alopecia. This comparison identifies a shared pathogenic layer—barrier disruption, innate immune activation, altered lipid or metabolite processing, and impaired anagen support—together with subtype-selective mechanisms, including immune-privilege collapse in alopecia areata, androgen–lipid–microbiome coupling in androgenetic alopecia, *Malassezia*-centered inflammatory ecology in seborrheic dermatitis, and infection/biofilm-driven injury with fibrosis in scarring disease. We further organize the available evidence into a 3-tier signaling cascade linking upstream microbial triggers to midstream NF-κB, JAK–STAT, PI3K/AKT, and Wnt/β-catenin integration and downstream outcomes such as stem-cell quiescence, dermal papilla dysfunction, vascular regression, immune-privilege loss, and fibrosis. We also evaluate probiotics, postbiotics, and engineered microbial systems, whose potential benefits may arise from coordinated anti-inflammatory, ecological, regenerative, and pro-angiogenic effects. However, translation is limited by methodological heterogeneity, weak causal evidence, strain stability, targeted delivery, manufacturing quality control, and regulatory uncertainty. Overall, this framework clarifies shared and subtype-specific roles of scalp dysbiosis and defines priorities for precision, microbiome-guided alopecia therapy.

## Introduction

Hair loss has become a common health issue, affecting approximately 50% of men and 25% of women worldwide [1]. Common types of hair loss include androgenetic alopecia (AGA), alopecia areata (AA), scarring alopecia, telogen effluvium, and anagen effluvium [2]. Common treatments encompass hair transplantation [3,4], medication [5], laser therapy [6–8], microneedling therapy [9–12], and autologous platelet-rich plasma stem cell therapy [13–15]. In recent years, hair loss has shown a trend toward increasing prevalence and earlier onset [16,17], imposing severe psychological stress on patients. This leads to negative emotions such as anxiety and low self-esteem, severely impacting personal quality of life and social activities.

The pathogenesis of hair loss remains complex and incompletely understood, potentially involving multiple factors including genetics [18–20], hormones [21,22], immunity [23], environment [24,25], and lifestyle [26]. Recent studies suggest that the scalp microbiome also markedly influences hair growth. As part of the human skin, the scalp harbors a unique microbial community that maintains scalp homeostasis [27]. Disruption of this balance may contribute to the onset and progression of hair loss [28]. Overgrowth or depletion of specific microbial strains can impair scalp barrier function, exacerbate inflammatory responses, and alter the follicular microenvironment, thereby disrupting the hair growth cycle [28–31]. In parallel, advances in organ-on-chip models, nanoscale quorum-sensing modulation, and marine-derived bioactive degradants are providing new experimental and engineering tools for investigating host–microbe interactions and developing targeted interventions for inflammation-associated follicular disorders [32–34]. Together, these findings support the scalp microbiome as both a mechanistic contributor to hair loss and a potential therapeutic target.

**Fig. 1.**
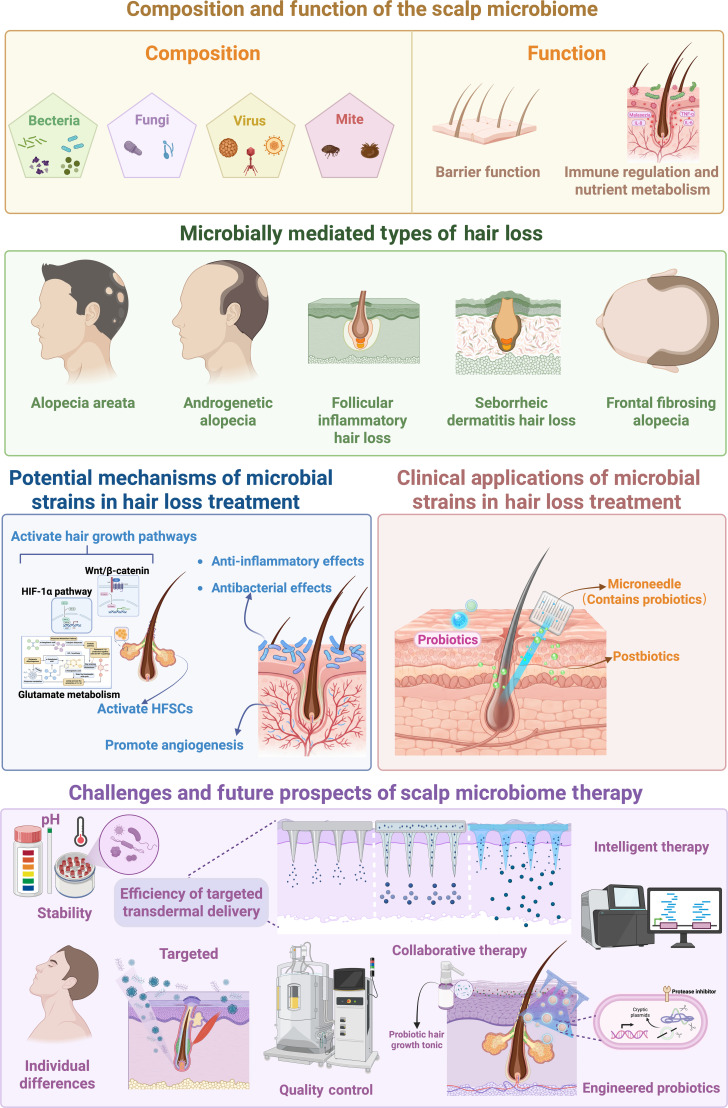
The scalp microbiome–hair axis: Mechanisms and therapeutic translation.

Accordingly, this review moves beyond a descriptive catalog of microbiome alterations and seeks to establish a clinically relevant and mechanistically coherent framework for the scalp microbiome–hair axis (Fig. 1). We first summarize the ecological composition and physiological functions of the scalp microbiome. We then compare dysbiosis signatures across major alopecia subtypes to distinguish shared pathological processes from subtype-specific patterns. Importantly, scalp dysbiosis should not be viewed as an isolated local event; emerging evidence suggests that cross-talk among microbial niches, environmental exposures, and host signaling pathways can destabilize microbial homeostasis and reshape the follicular microenvironment [35–37]. On this basis, we integrate upstream microbial triggers, host receptors, inflammatory mediators, metabolic alterations, stem-cell programs, and angiogenic responses into a unified signaling network linking dysbiosis to follicular dysfunction and hair-loss outcomes. We further reassess probiotics, postbiotics, and engineered microbial systems as therapeutic tools, emphasizing their potential synergistic actions rather than isolated strain-specific effects. Finally, we examine translational bottlenecks spanning formulation, targeted delivery, manufacturing, quality control, and regulation, and outline practical priorities for personalized, microbiome-guided treatment development. Within this translational landscape, sustainable nanoemulsions and phytochemical-based antimicrobial nanosystems represent promising next-generation topical platforms for restoring scalp microbial balance, improving local delivery, and reducing disease recurrence [38,39].

## The Scalp Microbiome: Composition, Function, and Dysbiosis in Hair Loss

### Composition of the scalp microbiome

**Fig. 2.**
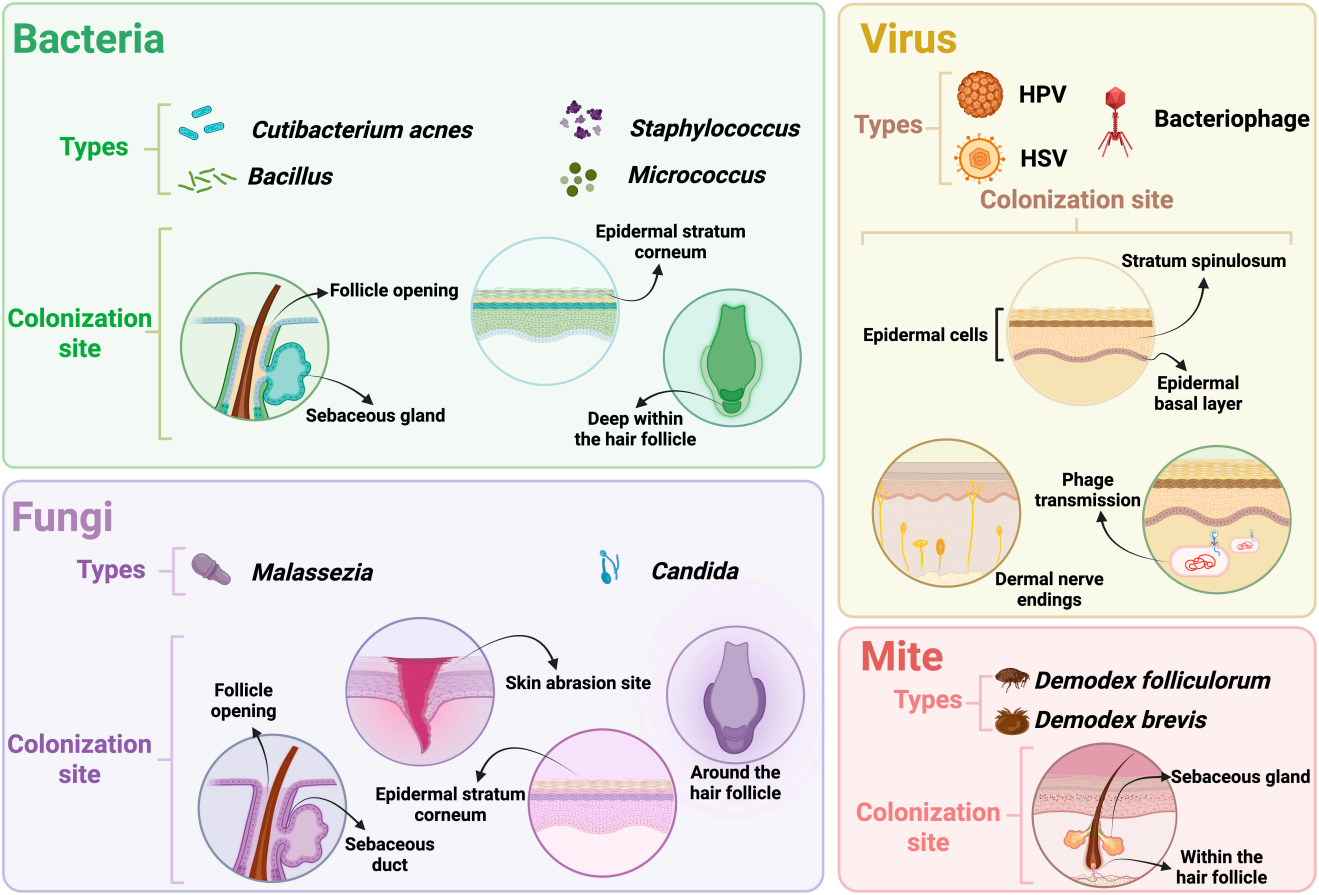
Schematic diagram of scalp microbiome colonization.

The scalp microbiome is a complex and dynamic ecosystem whose composition and function are crucial for scalp health (Table 1 and Fig. 2). Microbial density on the scalp reaches as high as 10^3^ to 10^5^ colony-forming units (CFU)/mm^2^, primarily comprising diverse microorganisms including bacteria, fungi, viruses, and parasites [40,41].

**Table 1.** Overview of the composition and distribution of the scalp microbiome

Category	Representative species	Colonization site	Ref.
Bacteria	*Cutibacterium acnes*	Sebaceous glands and ducts, deep within hair follicles	[28]
	*Staphylococcus*	Epidermal stratum corneum, around hair follicle openings	[42]
	*Bacillus*	Epidermal stratum corneum, around hair follicle openings, middle segment of sebaceous gland ducts	[43]
	*Micrococcus*	Epidermal stratum corneum	[44]
Fungi	*Malassezia* spp.	Epidermal stratum corneum, hair follicle orifices, sebaceous gland ducts	[45]
	*Candida* spp.	Epidermal lesions, perifollicular areas (pathological conditions)	[46]
Viruses	Human papillomavirus (HPV)	Basal layer of the epidermis, within spinous layer cells (latent infection)	[48]
	Herpes simplex virus (HSV)	Dermal nerve endings, epidermal cells (active phase)	[48]
	Various bacteriophages	Maintains symbiotic equilibrium with host bacteria, predominantly existing in an integrated state (prophage)	[49]
Mites	*Demodex folliculorum*	Within hair follicles (multiple clusters)	[40]
	*Demodex brevis*	Within sebaceous glands	[41]

The bacterial community is dominated by *Propionibacterium* (such as *Cutibacterium acnes*) and *Staphylococcus* (such as *Staphylococcus epidermidis*), which together account for over 90% of the total bacterial gene sequences on the scalp [28]. The *C. acnes* colonizes human hair follicles and sebaceous glands as a strict anaerobe. It releases free fatty acids through the metabolism of sebum secreted by sebaceous glands, maintaining a weakly acidic environment on the scalp surface [42]. When scalp hair follicles become blocked, sebum cannot be expelled normally, creating an oxygen-deprived environment within the follicle. This accelerates the overgrowth of *C. acnes*, leading to the accumulation of released free fatty acids and causing damage to the scalp barrier [43]. The latter is nonpathogenic on healthy scalp surfaces and even produces antimicrobial substances that inhibit the growth of surface pathogens. However, when the body’s immunity declines or mucosal integrity is compromised, it can trigger or exacerbate disease [44].

*Malassezia species* (such as *Malassezia restrictiva* and *Malassezia sphaerica*) are the most abundant fungi on the scalp [45]. *Malassezia* primarily survives and proliferates on human skin by secreting esterases that break down sebum produced by sebaceous glands [46]. Excessive secretion of *Malassezia* lipase and phospholipase hydrolyzes skin triglycerides, releasing unsaturated fatty acids such as oleic acid and arachidonic acid. This disrupts the barrier function of the stratum corneum, induces keratinocytes to produce pro-inflammatory cytokines, triggers skin inflammatory responses and desquamation, alters scalp pH, and damages the scalp barrier—all contributing factors to scalp inflammation [47,48].

Viruses (such as adeno-associated viruses and human papillomaviruses) and parasites (such as *Demodex folliculorum*) also play important roles in the scalp microbiome. These microorganisms maintain scalp microbial balance by consuming scalp secretions, supporting metabolism, and regulating the immune system [49]. When this equilibrium is disrupted, it may lead to issues like dandruff and hair loss.

### Function of the scalp microbiome

The scalp microbiome plays multiple critical roles in maintaining scalp health, primarily through barrier function, immune regulation, and nutrient metabolism. First, the microbiome helps maintain the integrity of the scalp barrier by forming biofilms and secreting antimicrobial peptides. For instance, resident flora like *S. epidermidis* and *C. acnes* suppress pathogenic microorganism colonization and prevent their overgrowth, thereby protecting the scalp from external pathogens [50]. Additionally, these microbes metabolize scalp secretions (e.g., sebum) to produce antimicrobial fatty acids, further enhancing barrier function [28]. Importantly, it is crucial to recognize the dual nature of many resident scalp microbes, which can oscillate between beneficial commensals and opportunistic pathogens depending on host–microenvironment interactions. A quintessential example is *C. acnes*. While it acts as a protective commensal maintaining homeostasis on a healthy scalp, specific microenvironmental alterations—such as excessive sebum secretion or an anaerobic environment caused by follicular blockage—can trigger its overproliferation. Under these dysbiotic conditions, *C. acnes* transitions into a pathogenic state, driving local inflammatory responses and contributing to the pathogenesis of scalp disorders like seborrheic dermatitis and folliculitis.

Secondly, the scalp microbiome regulates local immunity by balancing tolerance and defense. In a healthy state, commensal microbes prime the host immune system to defend against pathogens without causing destructive inflammation. However, during dysbiosis, this balance breaks down. The resulting immune overactivation or suppression can trigger scalp disorders, including dandruff and hair loss [51]. For example, the overgrowth of *C. acnes* strongly activates Toll-like receptor 2 (TLR2) on keratinocytes and immune cells. This induces a high release of pro-inflammatory cytokines, such as tumor necrosis factor-α (TNF-α), interluekin-6 (IL-6), and IL-8 [52]. Similarly, *Malassezia* overgrowth induces scalp inflammation [46], and *Staphylococcus aureus* colonization exacerbates abnormal immune responses by producing superantigens [53]. Ultimately, these microbial shifts convert normal immune defenses into chronic perifollicular inflammation, which disrupts the hair growth cycle.

Beyond the aforementioned dual roles, the scalp microbiome also plays an important role in nutrient metabolism. Microorganisms break down scalp secretions and keratinocytes, providing essential nutrients for both themselves and the host [28]. For instance, *Propionibacterium* species can break down triglycerides in sebum into free fatty acids. These fatty acids not only provide energy for microbes but can also be utilized by scalp cells to participate in cellular metabolism and repair processes [54,55]. Furthermore, microbial metabolites (such as short-chain fatty acids) may further optimize the scalp microenvironment by regulating local pH levels, thereby promoting follicular health [56].

In summary, the scalp microbiome maintains microecological balance and health through multiple mechanisms including barrier function, immune regulation, and nutrient metabolism. However, when the microbiome becomes imbalanced, these functions may be disrupted, leading to the development of scalp disorders. Therefore, in-depth research into the functions of the microbiome and its regulatory mechanisms is crucial for developing microbiome-based scalp health management strategies. In the future, precise modulation of microbial communities may offer novel approaches for preventing and treating scalp disorders.

### Scalp microbiome dysbiosis in hair loss: Subtype-specific alterations and cross-subtype patterns

Alterations in microbial density and diversity, enrichment of opportunistic taxa, loss of protective commensals, and shifts in the scalp physicochemical niche can collectively trigger barrier injury, inflammatory activation, and disruption of the hair cycle [49] (Table 2). Importantly, the available studies should not be read as isolated lists of taxa. When compared horizontally, they suggest a shared pathobiological logic: alopecia-associated dysbiosis usually features expansion of inflammation-amplifying organisms or metabolites, contraction of barrier-supportive and colonization-resistant communities, and progressive coupling between microbial imbalance and host programs such as innate immune activation, altered lipid handling, immune privilege collapse, and eventually fibrosis. At the same time, each alopecia subtype appears to emphasize a different dominant axis—immune privilege failure in AA, miniaturization-associated lipid–inflammation coupling in AGA, infection-driven neutrophilic inflammation in folliculitis/scarring disorders, and *Malassezia*–sebum–barrier loops in seborrheic dermatitis-related hair loss. This cross-subtype perspective is essential for distinguishing common therapeutic targets from subtype-specific intervention windows.

**Table 2.** Differential microbial abundance across various types of hair loss

Hair loss type	Microbes with increased abundance	Microbes with decreased abundance	Ref.
AA	*Cutibacterium acnes*, *Actinobacteria*, *Firmicutes*, *Corynebacterium*, *Prevotella*, *Acmannia mucilaginosa*	*Staphylococcus caprae*, *Staphylococcus epidermidis*, *Bacillus*, *Streptococcus*	[63,75]
AGA	*Cutibacterium acnes*, *Malassezia*, *Pseudomonas maltophilia*	*Staphylococcus epidermidis*, *Burkholderia* spp.	[71,152]
Folliculitis-related hair loss	*Staphylococcus aureus*, *Cutibacterium acnes*	No clear reduction in a single microbial community; overall microbial imbalance	[72,73]
Seborrheic dermatitis-related hair loss	*Staphylococcus* spp., *Malassezia* spp., *Exophilus* spp., *Bacillus subtilis*, *Bacillus* spp.	*Corynebacterium*, *Cutibacterium acnes*, *Aspergillus*	[77–79]
Fibrous alopecia	*Staphylococcus* spp., *Deuteromycota*	*Actinobacteria (Bacillus genus)*	[83,84]

The characterization of scalp microbial communities relies on a range of complementary techniques (Supplementary Materials). Traditional microbiological methods involve culturing scalp samples on selective media to isolate and identify microorganisms through colony morphology and biochemical reactions, though over 70% of scalp microorganisms remain nonculturable [57]. Biochemical analysis methods, such as matrix-assisted laser desorption/ionization time-of-flight mass spectrometry, enable rapid strain identification through protein fingerprint spectra [58]. Culture-independent molecular approaches have greatly expanded our understanding: 16S rRNA gene sequencing reveals community diversity and composition by amplifying conserved bacterial gene regions [59], while quantitative PCR enables precise quantification of specific target strains [60]. Together, these methods have revealed marked microbial alterations across different types of hair loss, as detailed below.

#### Alopecia areata

AA is a common autoimmune disorder characterized by nonscarring patches of hair loss on the scalp and other body areas. Its complex pathogenesis is often associated with depression, anxiety, vitiligo, and other autoimmune diseases, affecting approximately 2% of the global population [61,62]. Current research suggests that AA development is closely linked to compromised immune privilege, genetic susceptibility, and environmental triggers.

A U.S. study on pediatric AA patients revealed markedly elevated abundance of *Actinobacteria* and specific bacterial phyla within their scalp microbiome [62]. However, other studies present conflicting findings—some indicate no marked difference in microbial biodiversity between AA patients and healthy controls, though *Clostridium* abundance shows a decreasing trend [63]. Furthermore, analysis of the scalp microbiome in AA patients revealed markedly higher microbial diversity compared to healthy controls. This included an upward trend in *Propionibacterium* abundance and a marked decrease in *Staphylococcus* abundance, suggesting that *Staphylococcus* dysbiosis may contribute to the pathological process of AA [63]. De Pessemier et al. [64] further revealed that *Prevotella copri* and *Akkermansia muciniphila* abundances were markedly elevated in the subcutaneous tissue of AA patients compared to healthy controls. Notably, the expression pattern of *P. copri* mirrors the pathogenic mechanisms observed in rheumatoid arthritis, suggesting that this genus may activate hair-related autoimmune disorders through similar pathways. Meanwhile, the increased abundance of *A. muciniphila*, a strict anaerobe, suggests that the microenvironment surrounding hair follicles in AA patients may be hypoxic. This altered metabolic microenvironment may be potentially associated with the loss of follicular immune privilege [64].

In 2022, Won et al. conducted 16S rRNA amplicon metagenomic analysis to compare scalp bacterial microbiomes among young Chinese patients with mild and severe AA versus healthy controls. They found *Streptococcus caprae* abundance showed a marked negative correlation with AA disease progression, suggesting that reduced abundance of this genus may predict worsening clinical outcomes in AA. Concurrently, *Actinobacteria*—including *Corynebacterium* and *C. acnes*—exhibited markedly elevated abundance in the scalps of severe AA patients [65]. Conversely, Juhasz et al. [66] documented increased Clostridia (another phylum within the Firmicutes domain) on the scalps of AA patients.

Collectively, current evidence supports AA as a disorder in which microbiome alterations are most likely to function as immune modifiers rather than sole initiators. The recurring signals across studies—increased *Actinobacteria/Cutibacterium*-associated signatures, altered *Staphylococcus* balance, and enrichment of taxa linked to mucosal immune programming or anaerobic metabolism—are consistent with a model in which dysbiosis lowers the threshold for perifollicular immune activation and contributes to the collapse of follicular immune privilege. In this framework, microbial signals may interact with genetic susceptibility and autoimmune priming to convert a reversible inflammatory state into persistent follicular attack. This possibility is especially relevant for refractory AA and relapse-prone disease, where microbiome-mediated persistence of local inflammatory tone may partly explain incomplete response after immunosuppression alone.

#### Androgenetic alopecia

AGA is a common hair loss disorder characterized by follicular miniaturization, manifesting as progressively shorter, finer hair strands that may eventually fall out [67–69]. Recent studies investigating the involvement of specific bacterial strains in AGA mechanisms have provided new insights into the pathology of this condition.

Ho et al. conducted a 16S rRNA gene sequencing study on Asian males in Singapore to investigate the distribution characteristics of follicular microbiota in AGA patients. Results revealed marked differences in relative abundance of genus-level microbiota in miniaturized follicles on the vertex of AGA patients compared to occipital control (CO) and crown control (CV) follicles (*P* < 0.05). Notably, no marked differences in microbial abundance were observed when comparing nonminiaturized follicles from AGA patients with healthy controls [70]. This finding suggests that the microbial community characteristics of miniaturized follicles are closely associated with microbial species specifically colonizing follicles during the miniaturization process in AGA patients. Additionally, researchers detected a markedly elevated proportion of *C. acnes* in miniaturized hair follicles of AGA patients, accounting for 43.3%, compared to only 7.79% in nonminiaturized follicle samples. This was accompanied by up-regulation of innate immune response genes such as TLR2 and DEFB1, suggesting that this bacterium promotes follicular miniaturization by activating inflammatory pathways. The study also revealed specific microbial distributions across different anatomical regions of the hair follicle: *Burkholderia* spp. predominated in the middle region, while the lower region exhibited higher microbial diversity. Immune gene expression showed a direct correlation with bacterial abundance [70]. Through microbiome analysis, this research provides novel microbiological evidence for the pathogenesis of AGA, suggesting that specific microbial colonization in miniaturized hair follicles may contribute to the pathological process of AGA.

In 2021, Suzuki et al. studied Japanese male AGA patients and found an imbalance in scalp microbiota characterized by increased *C. acnes* abundance and decreased *Bacillus* spp. Additionally, elevated levels of triglycerides and palmitic acid were detected in sebum, with *Malassezia restrictiva* abundance positively correlated with palmitic acid content. This suggests that the fungus may utilize palmitic acid for proliferation and exacerbate inflammation [71].

Taken together, AGA-associated dysbiosis is best interpreted as a miniaturization-coupled ecological remodeling process rather than a nonspecific change in scalp flora. AGA appears to enrich for a high-sebum, pro-inflammatory follicular niche in which *C. acnes, Malassezia,* and lipid metabolites reinforce one another. These microbial changes are unlikely to replace the central roles of androgen signaling and genetic predisposition, but they may help explain interindividual heterogeneity in inflammation, progression rate, treatment responsiveness, and symptoms such as itch or seborrhea. Mechanistically, AGA therefore provides the clearest example of a scalp microbiome–endocrine–metabolic axis.

#### Inflammatory hair loss in follicles

Folliculitis-related hair loss is a collective term for disorders characterized by follicular inflammation that can progress to follicular destruction and permanent scarring alopecia. These conditions occupy the extreme end of the scalp microbiome–hair axis, where microbial invasion or dysbiosis is tightly coupled to overt neutrophilic inflammation, tissue injury, and irreversible architectural damage. The most common type is folliculitis decalvans, a neutrophilic primary scarring alopecia typically presenting with scalp papules, pustules, erythema, yellow crusts, and tufted hairs, often accompanied by itching, pain, or burning [72]. Other entities include acne keloidalis nuchae and drug-induced folliculitic alopecia [73–75]. In these disorders, *S. aureus* colonization, biofilm persistence, and host susceptibility likely sustain a self-reinforcing loop of follicular damage, neoantigen exposure, and chronic inflammation. Compared with nonscarring alopecias, this group highlights the downstream end of the pathogenic cascade, where delayed microbial control may culminate in stem-cell loss and fibrosis.

#### Seborrheic dermatitis-associated hair loss (D/SD)

D/SD refers to nonscarring hair loss closely associated with seborrheic dermatitis. Its core pathogenesis involves local inflammation, abnormal sebum secretion, and microbial imbalance—especially *Malassezia*-centered ecological shifts—that indirectly impair follicular function [76]. Rather than causing immediate follicular destruction, this condition mainly disrupts the hair cycle through chronic perifollicular inflammation, barrier fragility, itch–scratch amplification, and altered lipid metabolism, manifesting as scalp erythema, greasy scaling, pruritus, and diffuse shedding.

The onset of D/SD is not attributable to a single factor but rather results from the combined effects of genetic, environmental, immune, and microbial factors. Current research indicates that an imbalance in the scalp microbiome plays a pivotal role in D/SD pathogenesis, with the most marked dynamic changes observed in *Malassezia*, *Aspergillus*, *Staphylococcus*, and *Pseudomonas* [77]. Lin et al. demonstrated that fungal diversity on the scalps of D/SD patients was markedly lower than that in healthy controls. *Malassezia* dominated the fungal community in D/SD patients, with its density positively correlated to disease severity. This fungus triggers inflammatory responses and invades hair follicles, leading to folliculitis [78]. Conversely, *Aspergillus* abundance was markedly lower in patients than in healthy controls. Regarding bacterial communities, *Staphylococcus* abundance on the scalps of patients was markedly higher than that in healthy controls, with its overgrowth closely associated with epidermal barrier damage [79]. Conversely, *Pseudomonas* abundance was markedly lower in patients than in controls [77]. Because of their marked differences between the 2 groups, these 4 microorganisms hold potential as diagnostic and prognostic biomarkers for D/SD.

Pathologically, *Malassezia* breaches the skin barrier in susceptible individuals by metabolizing fatty acids in sebum or releasing immunostimulatory metabolites, thereby triggering inflammation. *Staphylococcus* exacerbates epidermal barrier disruption, forming a vicious “microbiome–inflammation” cycle [79]. Host intrinsic factors such as sebum secretion volume and composition (e.g., palmitic acid levels) can modulate *Malassezia* activity, further driving disease progression [80,81]. Notably, *Malassezia* abundance correlates directly with itch severity [79], while bacterial communities like *Bacteroides* and *Klebsiella* are more closely associated with D/SD-related bodily pain symptoms [81]. Clinically, selenium disulfide shampoo can augment ketoconazole therapy by restoring microbial balance, reducing recurrence risk [82]. Future research must elucidate the mechanisms underlying microbial interactions with the scalp microenvironment (e.g., pH and lipid metabolism) to provide theoretical foundations for developing precision therapies targeting microbial regulation.

#### Leaf-forehead fibrous alopecia

Leaf-forehead fibrous alopecia typically refers to central centrifugal scarring alopecia, a progressive, scarring form of hair loss that begins at the central scalp region and gradually expands outward in a centrifugal pattern. Its core pathological feature involves destruction of hair follicles accompanied by abnormal fibrosis, ultimately leading to permanent hair loss [83,84]. Its pathogenesis involves not only genetic and autoimmune factors but also an imbalance in the scalp’s microbial community. This imbalance may indirectly promote proliferation and destruction of the fibrous tissue surrounding hair follicles by triggering or sustaining local inflammatory responses, ultimately leading to scar formation and permanent hair loss [84]. A preliminary study on central centrifugal cicatricial alopecia revealed distinctive bacterial community profiles in patient scalps compared to healthy controls, most notably marked by markedly elevated relative abundance of *Bacillus* species [85]. These common skin bacteria, possessing pro-inflammatory potential, may contribute to disease progression by inducing or exacerbating the local inflammatory environment. Notably, the study did not identify marked differences in fungal communities or overall bacterial composition (excluding *Bacillus*) on patient scalps. This suggests that the disorder may be associated with dysregulation of specific bacterial groups rather than comprehensive disruption of the entire microbiome.

#### Cross-subtype comparison of scalp microbiome dysbiosis

**Fig. 3.**
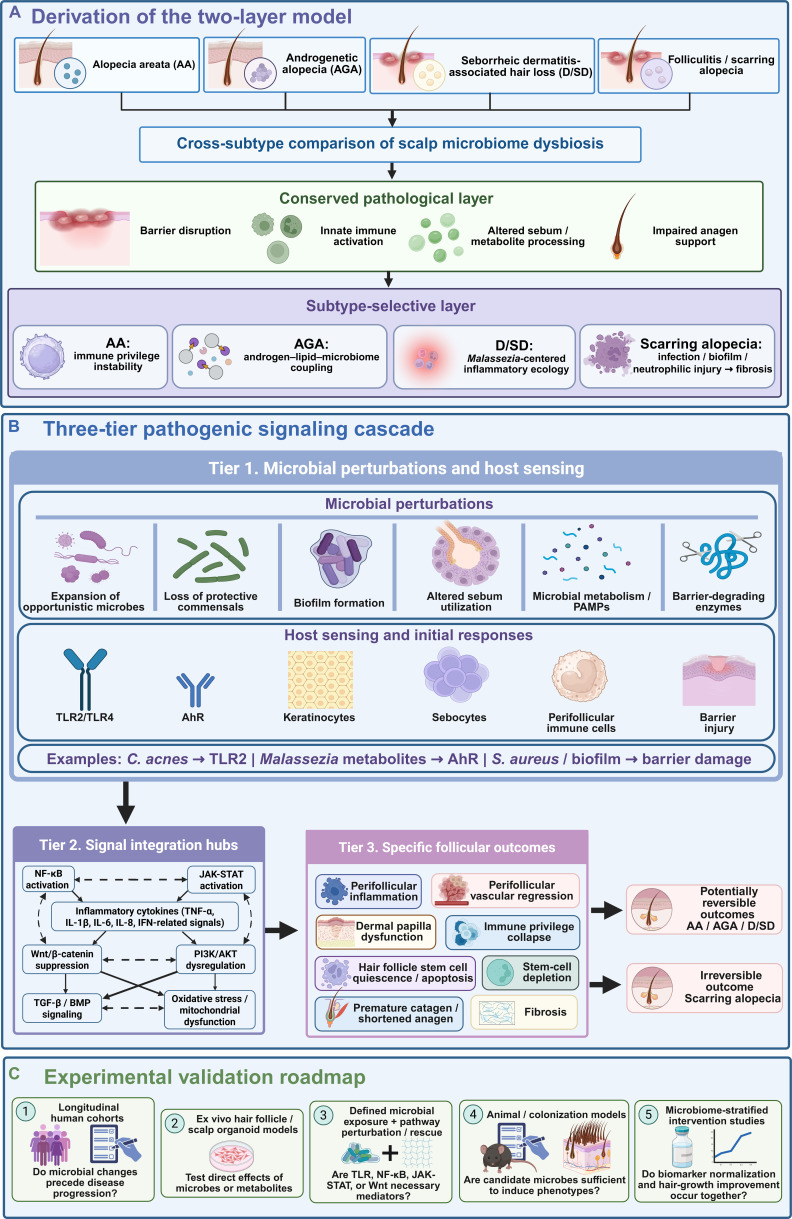
Conceptual framework of the scalp microbiome–hair axis: (A) evidence-derived 2-layer model, (B) 3-tier pathogenic signaling cascade, and (C) experimental validation roadmap.

Synthesizing the dysbiosis profiles described above [see the “Alopecia areata”, “Androgenetic alopecia”, “Inflammatory hair loss in follicles”, “Seborrheic dermatitis-associated hair loss (D/SD)”, and “Leaf-forehead fibrous alopecia” sections], several convergent and divergent patterns emerge across alopecia subtypes, enabling a more systematic understanding of the microbiome–hair loss nexus (Fig. 3). Despite converging evidence linking scalp dysbiosis to the pathogenesis of multiple alopecia subtypes, cross-study comparisons are substantially hampered by pronounced methodological heterogeneity across published research. As summarized in Table 3, variability in sampling protocols, sequencing strategies, and bioinformatic pipelines collectively contributes to inconsistent microbial signatures and limits the reproducibility of scalp microbiome findings.

**Table 3.** Methodological heterogeneity in scalp microbiome studies across major alopecia subtypes and its impact on result reproducibility

Alopecia subtype	Sampling heterogeneity	Sequencing and bioinformatic heterogeneity	Reported discrepancies in microbial signatures	Ref.
AA	Sampling sites include lesional patches, perilesional skin, and distant nonlesional scalp; swab sampling and punch biopsy methods are used interchangeably; patient subgroups vary widely in disease duration, severity, and concurrent immunosuppressive treatment status.	Bacterial 16S rRNA and fungal ITS sequencing are unevenly applied; few studies perform shotgun metagenomics; rarefaction depths and alpha-diversity calculation metrics lack standardization across datasets.	Controversy persists over whether *Cutibacterium* or *Staphylococcus* dominates lesional AA skin; fungal community dysbiosis in AA remains inconsistently reported across studies.	[169–172]
AGA	Sampling sites vary between vertex lesional areas and occipital nonlesional controls; sampling modalities include surface swabs, scalp scrapings, and hair follicle biopsies; presampling scalp washing and sebum collection protocols are inconsistent across cohorts.	16S rRNA variable regions range from V1–V3 to V3–V4; sequencing platforms include Illumina MiSeq, HiSeq, and NovaSeq; bioinformatic pipelines adopt QIIME 1, QIIME 2, or Mothur, with reference databases (Greengenes, SILVA) and clustering strategies (97% OTU vs. ASV) differing substantially between studies.	Some studies report *Cutibacterium acnes* enrichment in AGA lesions, whereas others show reduced abundance; findings on the relative abundance of *Staphylococcus* and *Malassezia* are inconsistent across different cohorts and sampling depths.	[57,173]
D/SD	Sampling targets seborrheic areas or normal scalp with large variation in baseline sebum secretion; sampling timing relative to shampoo use and scalp cleansing is not uniformly controlled.	Most studies prioritize fungal ITS sequencing with inconsistent bacterial profiling; different releases of the UNITE database yield discordant *Malassezia* species-level annotation; some studies supplement with GenBank-based reannotation to improve classification rates.	Debates persist over whether *Malassezia restricta* or *Malassezia globosa* is the dominant pathogenic species; the correlation between *Malassezia* abundance and clinical disease severity is not reproducible across populations.	[174–176]

First, *C. acnes* emerges as a convergent node across most nonscarring alopecia subtypes. Its abundance is elevated in both AA and AGA, where it has been shown to activate TLR2-mediated innate immune cascades and up-regulate defense genes such as DEFB1, driving perifollicular inflammation. However, its role is context-dependent: in seborrheic dermatitis-associated hair loss, *C. acnes* abundance is paradoxically reduced, suggesting that the pathogenic threshold of this organism may be modulated by the broader microbial community structure and host sebum metabolism rather than its absolute count alone.

Second, *Staphylococcus* species display a consistent but subtype-specific pattern of dysregulation. *S. epidermidis*, a key commensal whose antimicrobial metabolites maintain scalp homeostasis, shows reduced abundance in AA and AGA, effectively removing a natural check on pathogen expansion. Notably, in D/SD, the overall *Staphylococcus* genus abundance is markedly increased on patient scalps compared to healthy controls, but this enrichment likely reflects the expansion of pathogenic or opportunistic staphylococcal species rather than protective *S. epidermidis*, consistent with the observed exacerbation of epidermal barrier damage in these patients. In contrast, *S. aureus* is specifically enriched in folliculitis-related scarring alopecia, where it acts as a direct inflammatory driver through superantigen production and disruption of host immune homeostasis. This divergence highlights a fundamental distinction between immune-mediated (AA and AGA) and infection-driven (folliculitis) alopecia from a microbiological perspective.

Third, fungal dysbiosis—particularly the expansion of *Malassezia* species—provides a distinguishing signature for seborrheic dermatitis-associated and AGA-related hair loss, where lipid metabolism aberrations in sebum create a favorable niche for fungal overgrowth. By contrast, fungal community composition is relatively undisturbed in central centrifugal cicatricial alopecia, where bacterial (*Bacillus*) dysbiosis predominates. These data collectively suggest that integrating fungal and bacterial profiling may offer superior discriminative power for differential diagnosis compared to analyzing either kingdom in isolation.

Fourth, when viewed through the lens of inflammatory polarity, we propose that the alopecia subtypes can be broadly separated into 2 clusters: (a) Th1-driven autoimmune types (AA), characterized by enrichment of immunostimulatory bacteria such as *P. copri*, whose expression pattern mirrors pathogenic mechanisms observed in autoimmune disorders such as rheumatoid arthritis; and (b) innate immunity-driven types (AGA and D/SD), dominated by TLR2/TLR4-activating organisms and their lipid metabolites. Scarring alopecia may occupy an intermediate position, involving both *S. aureus*-driven innate activation and aberrant adaptive fibrotic responses.

Fifth, although data remain limited, findings in fibrosing/scarring alopecia suggest that microbiome signals may become particularly consequential when tissue repair is misdirected toward chronic inflammation and fibrosis. Unlike AGA or D/SD, in which dysbiosis mainly perturbs cycling and miniaturization, scarring alopecias represent failure of regenerative recovery. The enrichment of selected pro-inflammatory taxa, together with architectural destruction of follicles, supports the hypothesis that microbial cues may help maintain fibro-inflammatory circuits once disease is established. This concept may also be relevant for other difficult-to-treat or atypical alopecias, including congenital or structurally predisposed hair disorders, for which direct microbiome data are currently lacking. In these conditions, the microbiome may not be the primary cause but may still shape disease penetrance, inflammatory flares, and therapeutic responsiveness by modulating barrier resilience and immune set points.

Overall, the currently available evidence supports a 2-layer model of scalp microbiome involvement in hair loss. At the conserved layer, multiple alopecia subtypes converge on dysbiosis-associated barrier disruption, innate immune activation, altered sebum or metabolite processing, and impaired support for anagen maintenance. At the subtype-selective layer, AA is dominated by immune privilege instability, AGA is dominated by androgen-lipid-microbiome coupling, D/SD is dominated by *Malassezia*-centered inflammatory ecology, and folliculitis/scarring alopecia is dominated by infection-biofilm-neutrophilic injury progressing toward stem-cell depletion and fibrosis. This comparative framework helps explain why no single microbial intervention is likely to fit all alopecia types and why future studies must stratify patients by both clinical phenotype and microbiome state.

The 2-layer model was derived through a horizontal comparison of microbial and host-response patterns reported across the major alopecia subtypes reviewed above. Features repeatedly observed in more than one subtype—including barrier impairment, innate immune activation, altered sebum or microbial metabolite processing, and reduced support for anagen maintenance—were assigned to the conserved layer. In contrast, mechanisms showing preferential association with a particular clinical phenotype were assigned to the subtype-selective layer, including immune privilege instability in AA, androgen–lipid–microbiome coupling in AGA, Malassezia-centered inflammatory ecology in D/SD, and infection–biofilm–neutrophilic injury in folliculitis/scarring alopecia. This classification is therefore based on convergence and divergence across published studies rather than on a single dataset.

Notably, because the underlying evidence supporting this framework—including the microbial dysbiosis signatures and the 2-layer model summarized above—is predominantly derived from heterogeneous, cross-sectional case–control studies in human populations, it can only demonstrate correlative associations rather than definitive causality. Consequently, this framework should be interpreted as a testable synthesis rather than a conclusive causal classification. Whether the enrichment or depletion of specific microbial taxa directly drives hair follicle dysfunction and hair loss, or instead occurs as a secondary consequence of the altered follicular microenvironment, remains to be rigorously verified by prospective longitudinal cohorts, germ-free animal colonization experiments, and targeted microbial intervention trials. The mechanistic pathogenic cascade elaborated in the subsequent section represents an integrative hypothetical framework constructed based on current evidence, and its complete causal chain still requires further experimental validation.

#### From microbial dysbiosis to follicular dysfunction: The pathogenic signaling cascade

The dysbiosis patterns described above converge, through distinct but overlapping routes, on a common endpoint: disruption of the hair follicle microenvironment and derailment of the growth cycle. To move beyond cataloguing microbial shifts and toward a mechanistic understanding of how these shifts cause follicular pathology, it is useful to trace the signaling cascade from the initial microbial trigger through intracellular signal integration to the final effector responses that determine follicular fate. This cascade can be broadly organized into 3 tiers.

At the upstream tier, dysbiotic microorganisms and their products generate pathogen-associated molecular patterns and damage-associated molecular patterns that engage pattern recognition receptors (PRRs) on keratinocytes, Langerhans cells, and perifollicular macrophages. Lipoteichoic acid from gram-positive bacteria activates TLR2 [86], while lipopolysaccharide from gram-negative bacteria engages TLR4, together initiating local innate immune signaling. In parallel, *Malassezia*-derived indole derivatives and unsaturated fatty acids stimulate the aryl hydrocarbon receptor (AhR) on keratinocytes and sebocytes, coupling microbial lipid metabolism to host inflammatory and sebaceous programs [47,48]. The physical barrier itself is also directly compromised: extracellular proteases secreted by *S. aureus* degrade tight junction proteins in the epidermal and follicular epithelium [53], breaching the structural barrier and exposing subepithelial immune sentinels to microbial antigens. Importantly, the nature and intensity of these upstream triggers differ by alopecia subtype—*C. acnes*-driven TLR2 activation predominates in AA and AGA [65,70,71], *Malassezia*-AhR signaling is central to D/SD [77,78], and *S. aureus*-mediated barrier breach is the hallmark of folliculitis-related scarring alopecia [72]—consistent with the subtype-selective dysbiosis patterns outlined in the “Cross-subtype comparison of scalp microbiome dysbiosis” section.

Once triggered, these diverse upstream inputs converge at the midstream signal integration tier on a limited set of intracellular hubs. The nuclear factor κB (NF-κB) pathway functions as a master inflammatory switch, integrating TLR2/4 signals to drive transcription of pro-inflammatory cytokines including TNF-α, IL-6, and IL-1β, and to promote downstream Th17 polarization [44]. In AA specifically, JAK-STAT signaling—particularly the JAK1/2–STAT1/3 axis—amplifies interferon-γ responses that are central to the collapse of follicular immune privilege [23]. The PI3K/AKT pathway, which normally supports dermal papilla cell survival and anagen maintenance [87], may be suppressed under chronic inflammatory conditions, contributing to premature catagen entry. A particularly consequential cross-talk occurs between inflammatory signaling and the Wnt/β-catenin pathway: sustained NF-κB activity up-regulates Wnt inhibitors DKK1 and SFRP1 [88], while simultaneously activating the BMP/Smad axis that maintains hair follicle stem cell quiescence [89,90]. The net effect is a molecular brake on hair cycling—inflammatory signals do not merely damage existing follicles but actively suppress the regenerative machinery that would otherwise initiate new growth.

These midstream perturbations ultimately manifest at the downstream effector tier as a set of interconnected pathological outcomes that collectively determine whether hair loss is reversible or permanent. Suppression of Wnt/β-catenin coupled with activation of p53/p21 pushes hair follicle stem cells toward prolonged quiescence or apoptosis, while TGF-β/BMP4 up-regulation and FGF5 overexpression drive premature catagen induction [89,91,92]. Dermal papilla cells—the mesenchymal signaling center of the follicle—undergo functional decline through oxidative stress and mitochondrial dysfunction [93], losing their capacity to instruct follicular cycling. Simultaneously, perifollicular vasculature regresses as vascular endothelial growth factor (VEGF) expression is down-regulated [94,95], depriving the follicle of the nutrient and oxygen supply essential for the energy-intensive anagen phase. In autoimmune alopecia, this cascade extends to encompass the collapse of follicular immune privilege itself: major histocompatibility complex class I/II molecules are up-regulated on follicular epithelial cells, exposing melanocyte-associated autoantigens to CD8^+^ NKG2D^+^ T cells and converting transient perifollicular inflammation into persistent, targeted follicular attack. In scarring alopecias, the cascade progresses further still—chronic inflammation and stem-cell depletion trigger fibrotic replacement of the follicular unit, rendering hair loss irreversible.

The distinction between reversible and irreversible endpoints has direct therapeutic implications. In nonscarring conditions such as AA and AGA, the stem cell niche remains largely intact, meaning that interventions capable of silencing the inflammatory cascade and reactivating Wnt signaling retain the potential to restore cycling. In scarring conditions, the therapeutic window is narrower: once fibrosis has replaced the follicular architecture, even complete resolution of dysbiosis cannot regenerate lost follicles. This underscores the urgency of early microbial intervention in inflammation-prone scalp conditions and provides a mechanistic rationale for the therapeutic strategies discussed in the following section.

While this 3-tiered pathogenic cascade provides a compelling mechanistic model, its definitive causal sequence remains to be fully established. Moving forward, rigorous validation of this framework will require sequential testing of temporal association, biological sufficiency, mechanistic necessity, and therapeutic reversibility. First, prospective longitudinal cohorts with standardized scalp sampling should determine whether microbial alterations precede changes in barrier function, inflammatory signaling, and hair-cycle parameters. Second, ex vivo human hair follicles, scalp organoids, and reconstructed follicular skin models can be exposed to defined microorganisms, microbial consortia, or metabolites to test their effects on TLR/AhR activation, NF-κB and JAK–STAT signaling, Wnt/β-catenin activity, dermal papilla function, and hair-shaft elongation. Third, gnotobiotic or antibiotic-conditioned animal models can assess whether candidate organisms are sufficient to reproduce follicular phenotypes and whether genetic or pharmacological pathway blockade prevents these effects. Finally, microbiome-stratified intervention trials should determine whether correction of dysbiosis produces parallel normalization of molecular biomarkers and clinically meaningful hair-growth outcomes. Such studies would help distinguish causal drivers from secondary ecological changes.

## Potential Mechanisms of Microbial Strains in Hair Loss Treatment

**Fig. 4.**
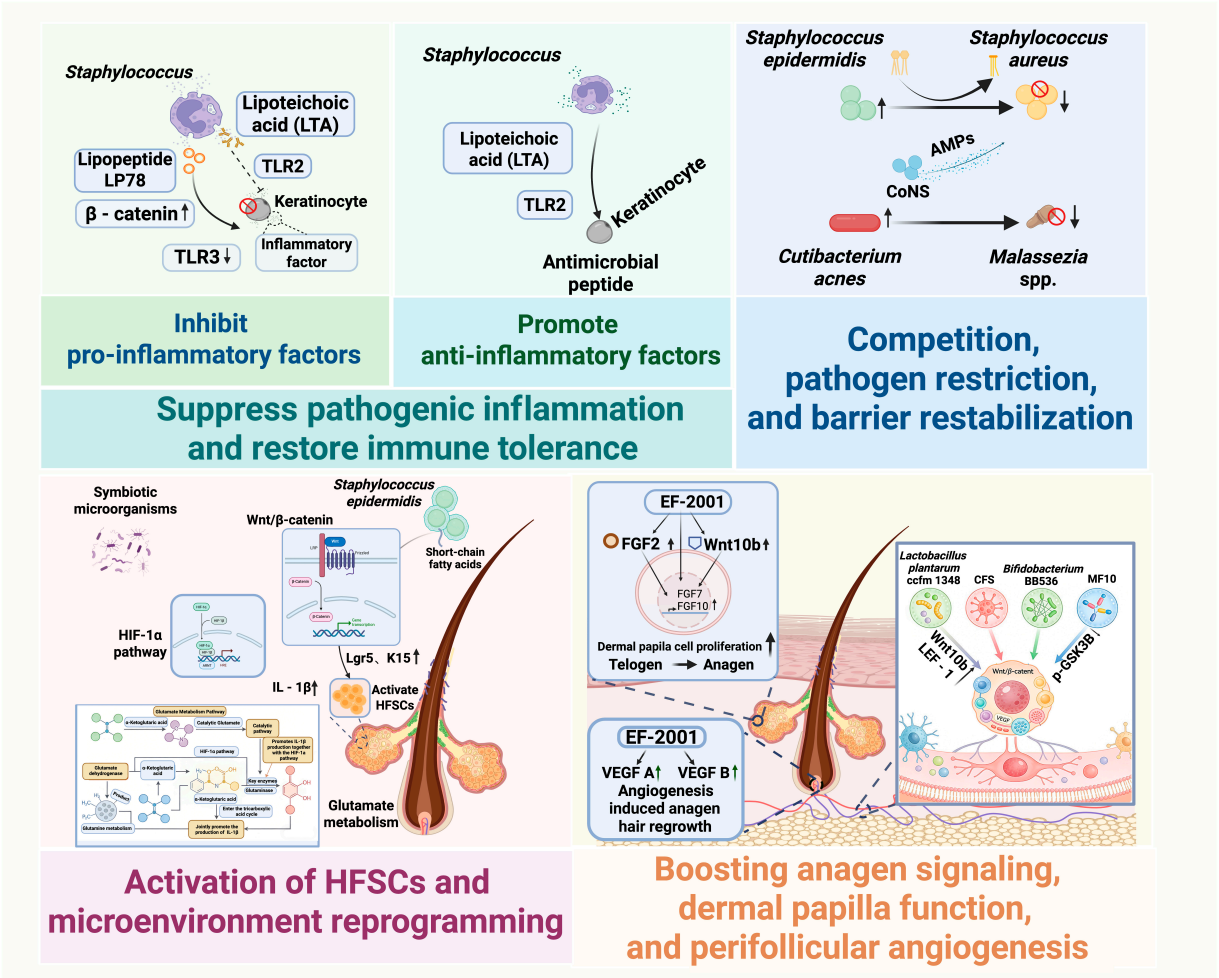
Potential mechanisms of scalp microbiotas in treating hair loss.

Building on the comparative dysbiosis patterns described above, the microbiome–hair axis can be organized as a directional network rather than a collection of disconnected observations. Upstream events include expansion of opportunistic microbes, loss of protective commensals, altered sebum utilization, biofilm formation, and release of immunogenic cell-wall components or metabolites. These signals are sensed by keratinocytes, sebocytes, perifollicular immune cells, and dermal papilla-associated cells through pattern-recognition pathways such as TLR2/TLR3 and related innate signaling modules, leading to production of TNF-α, IL-1β, IL-6, IL-8, interferon-related mediators, antimicrobial peptides, and oxidative stress responses [46,52,53]. Once this inflammatory threshold is crossed, downstream consequences include collapse of follicular immune privilege, altered stem-cell quiescence, impaired dermal papilla function, shortened anagen, perifollicular microvascular insufficiency, and, in severe cases, matrix damage and fibrosis. Beneficial microbes and microbial-derived products may interrupt this cascade at multiple levels simultaneously: they can restore ecological competition, reduce pathogen burden, dampen inflammatory signaling, rebalance metabolite pools, and reactivate pro-regenerative pathways such as Wnt/β-catenin, PI3K/AKT, and HIF-1α-linked programs. Therefore, the therapeutic question is not simply whether a strain promotes hair growth, but which node or combination of nodes within this pathogenic network it modulates (Table 4 and Fig. 4). Notably, although microbial metabolites are theoretically critical regulators of scalp physiology and follicular function, direct evidence linking scalp microbial metabolic products to hair growth modulation is still scarce in the existing literature. The metabolic layer of host–microbiome crosstalk in the scalp remains under-investigated relative to immune and barrier-related mechanisms, and warrants dedicated functional exploration in future studies.

**Table 4.** Function of the scalp microbiome for hair regeneration

Function		Description	Impact on hair health	Ref.
Barrier function	Antimicrobial peptide production	Microbiome secretes substances that inhibit pathogen growth	Enhanced immune defense of the scalp	[177]
	Biofilm formation	Microorganisms form a protective layer on the hair surface	Protecting the scalp and hair from external aggressors	[178]
	Defends against pathogens	Prevents colonization by harmful microorganisms	Reduces the risk of infections and scalp conditions	[179,180]
	Protect against ultraviolet radiation	Microorganisms produce compounds that absorb or deflect ultraviolet rays	Minimizing UV damage to scalp and hair	[181]
	pH regulation	Microbiome maintains a balanced pH environment	Prevents harmful microbial overgrowth	[30]
	Detoxification	Microbial breakdown of harmful substances	Reduce hair damage caused by toxins	[182]
Interacts with the scalp and microbiome	Immune regulation	Modulation of local immune responses	Reduce inflammation and allergic reactions	[183]
	Synergistic effects	Collaborates with the skin microbiome to enhance overall health	Promoting balance and health in the scalp environment	[184]
	Antagonism	Beneficial microorganisms suppress harmful microorganisms through resource competition	Maintaining microbial balance and health	[185]
	Metabolic exchange	Beneficial metabolic exchange between the scalp and hair follicle microbiome	Supports nutrient supply to the scalp	[184]
	Communication with host cells	Signaling to host cells to regulate growth and defense	Promoting follicular health	[93]
	Influencing sebaceous and sweat glands	Influences sebum production and formation, regulates sweat gland secretion	Controls sebum secretion while maintaining scalp moisture balance and hydration	[186,187]
Influences hair growth	Produces anti-inflammatory compounds	Promotes expression of anti-inflammatory factors	Reduce scalp irritation	[188]
	Inhibits pathogenic bacteria	Inhibits pathogenic bacteria by competing for nutrients in the scalp epidermis	Effectively alleviates inflammation, maintains hair follicle function, and improves hair loss symptoms	[99]
	Activates hair follicle stem cells	Initiates the transition of hair follicles from the resting phase to the growth phase	Promotes hair growth and reduces hair loss	[106,107]
	Activates hair growth pathways	Activate the Wnt/β-catenin signaling pathway	Promote proliferation and differentiation of hair follicle stem cells, extending the anagen phase	[89,90]
	Promotes angiogenesis	Promotes proliferation of vascular endothelial cells	Provides nutrients to hair follicles, promoting hair growth	[94,95]

### Suppression of pathogenic inflammation and restoration of immune tolerance

Inflammation is the most reproducible interface between microbial dysbiosis and hair loss. In both inflammatory and noninflammatory alopecia, dysbiosis can convert a physiologic surveillance state into persistent perifollicular damage by increasing pro-inflammatory cytokines, weakening immune tolerance, and amplifying tissue stress. From a mechanistic standpoint, 2 linked processes are especially important: first, suppression of excessive innate inflammatory output; and second, restoration of regulatory signals that prevent chronic immune-mediated injury. This dual framing is more informative than treating anti-inflammatory effects as a single undifferentiated mechanism.

#### Suppression of pro-inflammatory factors to limit follicular damage

Certain bacterial strains can alleviate inflammatory responses around hair follicles by suppressing the release of pro-inflammatory factors. Li et al. [96] discovered that the lipopeptide LP78 from *S. epidermidis* activates β-catenin, inhibits TLR3-mediated skin inflammation, and promotes wound healing. In the context of hair loss, this finding is important because it indicates that microbial molecules may simultaneously blunt inflammatory injury and preserve regenerative signaling. Likewise, lipoteichoic acid from *staphylococci* suppresses keratinocyte inflammatory responses through a TLR2-dependent mechanism, reducing injury-induced cytokine release [86]. Together, these studies suggest that selected microbial signals do not merely reduce inflammation in a nonspecific manner; rather, they may recalibrate the threshold at which the follicular unit interprets microbial cues as danger signals, thereby decreasing the probability that transient ecological disturbance evolves into chronic perifollicular inflammation.

#### Promotion of anti-inflammatory programs and immune retolerization

Beyond direct inhibition of pro-inflammatory mediators, beneficial microbes may also restore the regulatory arm of the local immune network. *Lactobacillus* strains have been reported to increase IL-10 production, rebalance Th1/Th2 responses, and attenuate skin inflammation [97]. Small molecules produced by *S. epidermidis* can activate protective TLR2 signaling in keratinocytes, inducing antimicrobial peptide expression while supporting barrier defense [98]. These observations are especially relevant to AA and chronic relapsing scalp inflammation, where durable control likely requires re-establishment of immune tolerance rather than short-term cytokine blockade alone. Thus, anti-inflammatory efficacy should be understood as the net result of suppressing harmful signals while rebuilding a permissive immunological environment for follicular cycling.

### Ecological competition, pathogen restriction, and barrier restabilization

Scalp health relies on dynamic ecological competition. Beneficial strains can protect hair follicles not only by secreting anti-inflammatory products, but also by occupying ecological niches, consuming key nutrients, acidifying the local milieu, producing bacteriocins, and limiting pathogen biofilms. *S. epidermidis* and other coagulase-negative *staphylococci* can inhibit *S. aureus* through free-fatty-acid production and antimicrobial peptides [99–104]. *Cutibacterium*-derived metabolites may also restrict competing organisms under specific ecological conditions [105]. In therapeutic terms, this means that microbial hair-growth promotion is often indirect: by re-establishing colonization resistance and barrier compatibility, beneficial microbes prevent repeated pathogen-triggered inflammatory flares that would otherwise damage the follicular unit. This ecological mechanism is likely to be particularly relevant in seborrheic dermatitis-associated shedding and folliculitis/scarring disorders, where microbial competition and biofilm control may decisively influence progression.

### Activation of hair follicle stem cells and regenerative microenvironment reprogramming

Hair follicle stem cells (HFSCs) located in the bulge region govern the transition from telogen to anagen and are highly sensitive to inflammatory, metabolic, and hypoxic cues. Dysbiosis can impair HFSC activation indirectly by maintaining cytokine-rich, oxidative, or metabolically hostile surroundings; conversely, beneficial microbes may restore a pro-regenerative microenvironment that permits HFSC proliferation and differentiation. This distinction is important because many microbiome-derived interventions are unlikely to act as classical mitogens in isolation, but rather as niche modulators that shift the local balance from stem-cell suppression to stem-cell competence.

Several lines of evidence support this concept. *S. epidermidis*-derived metabolites have been linked to activation of Wnt/β-catenin signaling and up-regulation of HFSC markers such as Lgr5 and K15, while reducing TNF-α [102,106,107]. Studies of skin microbiota-regulated regeneration further suggest that microbial cues can induce keratinocyte hypoxia signaling, activate HIF-1α-associated pathways, reshape glutamine metabolism, and ultimately support regenerative programs within the follicular niche [108]. Heat-killed *Enterococcus faecalis* EF-2001 likewise enhanced dermal papilla cell proliferation and induced expression of Wnt-related genes and growth factors including FGF2, FGF7, FGF10, VEGF-A, and VEGF-B [109]. Taken together, these data indicate that microbial interventions may act upstream of HFSC activation by coordinating inflammatory relief, metabolic rewiring, and growth-factor induction.

Importantly, these mechanisms are not independent. A regenerated follicular niche is more resistant to pathogen invasion, while reduced pathogen burden further lowers inflammatory stress and preserves stem-cell activity. This reciprocal reinforcement helps explain why multicomponent microbial interventions may outperform single-target strategies.

### Reinforcement of anagen signaling, dermal papilla function, and perifollicular angiogenesis

The Wnt/β-catenin axis is a central positive regulator of hair follicle morphogenesis, anagen entry, and HFSC differentiation [89–92]. However, its activity is embedded within a broader regenerative network that also includes PI3K/AKT signaling, growth-factor release, androgen-response modulation, matrix cell survival, and vascular support. Therefore, when evaluating microbiome-based therapies, activation of Wnt signaling should be interpreted as one component of a coordinated systems-level response rather than the only relevant endpoint.

Perifollicular angiogenesis is one of the most important downstream readouts of this coordinated response. Hair follicles require a dynamic microvascular supply of oxygen, nutrients, and signaling molecules, and anagen is normally accompanied by increased vessel number and caliber [94,95,110]. Microbial strains or postbiotics that up-regulate VEGF, protect dermal papilla cells, or improve the inflammatory-metabolic environment may therefore support hair growth through both direct follicular signaling and indirect vascular rescue.

Consistent with this model, *Lactobacillus fermentum* MF10 and fermented goji berry preparations promoted hair growth while modulating β-catenin-related signaling and VEGF levels [111]. Cell-free supernatant from *Bifidobacterium bifidum* BB536 repaired DHT-damaged human dermal papilla cells, increased VEGF secretion, activated Wnt10b/β-catenin/LEF-1 signaling, and suppressed DKK1 and GSK-3β [92]. *Lactobacillus plantarum* CCFM1348-fermented Thuja extract likewise enhanced dermal papilla proliferation, up-regulated β-catenin, and increased VEGF expression in vivo [112]. These studies support a unifying interpretation: microbiome-derived interventions promote anagen not through one linear pathway, but by converging on dermal papilla viability, stem-cell permissiveness, and vascular support.

Notably, microbiome-associated regulatory pathways do not act in isolation from well-established classical drivers of alopecia, including androgen signaling, follicular cycling dynamics, and hair follicle immune privilege regulation. Instead, microbial dysbiosis and these traditional pathogenic factors form a bidirectional, interconnected regulatory network. For instance, excess androgens shape scalp microbial community structure by boosting sebum production and remodeling the follicular microenvironment, while microbial metabolites and inflammatory mediators can, in turn, modulate local androgen metabolism, disrupt anagen maintenance, and impair follicular immune privilege. This pervasive reciprocal crosstalk makes it inherently difficult to quantify the relative biological contribution of each factor, or to definitively distinguish primary pathogenic drivers from secondary amplifiers. To date, there remains a lack of rigorous causal validation studies—such as germ-free animal models, standardized microbiota transplantation assays, and large-scale longitudinal interventional trials—that can disentangle the independent effects of the microbiome from classical endocrine and immune mechanisms, and clarify their respective weights in alopecia pathogenesis.

In summary, microbial strains demonstrate potential in promoting hair growth because their actions are inherently networked. Anti-inflammatory signaling preserves the follicular niche; ecological competition reduces repeated pathogen-triggered injury; metabolic and hypoxic reprogramming reactivate stem-cell competence; and Wnt/growth-factor/angiogenic pathways sustain anagen execution. This synergy is especially important for refractory and special alopecia types. In congenital hair disorders or structurally abnormal follicles, the microbiome is unlikely to be the primary initiating factor, but it may still modify barrier integrity, inflammation, and treatment tolerance. In chronic AA, recurrent folliculitis, and fibro-inflammatory alopecias, microbiome-targeted therapy may be most useful as a disease-modifying adjunct that reduces relapse drivers and improves the local regenerative set point. Future mechanistic studies should therefore move beyond single-strain/single-pathway claims and instead test how defined microbial consortia rewire the full scalp–hair axis under specific clinical phenotypes.

## Clinical Topical Application of Microbiotas for Hair Loss Treatment

In recent years, with the continuous advancement of research on the human skin microbiome, the close association between the scalp microbiome and hair follicle health has gradually been revealed. Hair loss treatment strategies centered on regulating scalp microbiome balance through specific microbial strains have entered the clinical stage, with some products even reaching market availability (Table 5). Their core advantage lies in fundamentally improving hair follicle survival conditions by restoring a healthy scalp microenvironment, thereby promoting hair growth.

**Table 5.** Clinical trials for microbial treatment of hair loss are currently underway

Hair loss treatment	Indication	Therapeutic agent	Intervention strategy	Phase	NCT identifier
AGA	Hair density, hair diameter	*Lactobacillus reuteri*	Apply a solution containing *Lactobacillus reuteri* powder to the affected area daily	Not applicable	ChiCTR2500105724
	Hair density, sebaceous gland contents, scalp dandruff	Beneficial bacterial freeze-dried powder	–	Not applicable	ChiCTR2400080391
	Hair density, changes in hair microbiome	HT-LM1020	Daily application of a hair growth agent containing 5% HT-LM1020, 0.3% menthol, 0.25% salicylic acid, and 0.2% panthenol	–	GIRB-21O29-ET
AA	Hair growth rate, side effects	*Candida albicans* antigen	Subcutaneous injection of *Candida albicans* antigen	Not applicable	NCT06564805
	Hair growth rate	Lactic acid solution	Microneedling combined with 1% lactic acid solution or vitamin D3 or triamcinolone acetonide for aa	Not applicable	NCT06327581
Hair loss	Hair thickness, hair growth rate, hair density	Biotin plant extract	Oral	Not applicable	NCT05972512
	Hair growth rate, hair density, hair cycle assessment	Fermented extract	Apply the essence directly to the scalp twice daily using a roll-on applicator	Not applicable	NCT07195487
	Hair growth rate	Lactic acid solution	Combined microneedling therapy with 1% lactic acid solution or vitamin D3 or triamcinolone acetonide for AA	Not applicable	NCT06327581
Hair loss	Hair thickness, hair growth rate, hair density	Biotin plant extract	Oral	Not applicable	NCT05972512
	Hair growth rate, hair density, hair cycle assessment	Fermented extract	Apply the essence directly to the scalp twice daily using a roll-on applicator	Not applicable	NCT07195487
	Dandruff, hair growth rate, changes in the hair microbiome	GMNL-653	Use GMNL-653 shampoo containing heat sterilization	–	–

Preparations containing specific live microbial strains hold potential value in treating hair loss and improving hair density. However, inherent challenges such as poor stability, limited scalp colonization capacity, and unclear long-term safety require further validation through larger-scale, rigorously designed clinical studies. Compared to live probiotics, postbiotics have emerged as a core direction in microbial therapy for hair loss intervention due to their superior practicality and safety profile.

Postbiotics specifically refer to nonliving microbial components or their metabolic products that benefit host health. They primarily include fermentation filtrates containing active ingredients such as amino acids, vitamins, peptides, and organic acids produced by probiotics (e.g., *Fermentum* and *Lactobacillus*), as well as specific metabolites like short-chain fatty acids (butyric acid and propionic acid), bacteriocins, and enzymes [113,114]. The mechanism by which postbiotics promote hair growth involves a series of complex biological regulations, including activation of key cellular signaling pathways, modulation of growth factor expression, influence on scalp microbiome balance, and reduction of apoptosis [115,116]. Research indicates that postbiotics can directly activate dermal papilla cells and key signaling pathways. Tang et al. discovered that a postbiotic herbal extract (*Cacumen platycladi*, CP) fermented by specific *L. plantarum* promotes proliferation of these cells by up-regulating β-catenin, a key factor in the Wnt/β-catenin signaling pathway, thereby advancing hair follicles into the anagen phase. Beyond activating core signaling pathways, postbiotics also modulate a series of growth factors closely associated with hair growth [112]. In animal studies, Tang et al. observed that CP increased VEGF expression in mouse dorsal skin, thereby improving blood supply and nutrient delivery around hair follicles. Concurrently, CP reduced the ratio of pro-apoptotic protein BAX to anti-apoptotic protein Bcl-2, indicating its potential to decrease follicular cell apoptosis and prolong follicular survival [112].

Another study also confirmed that heat-inactivated probiotic postbiotics composed of *Lacticaseibacillus paracasei* (GMNL-653) can up-regulate mRNA expression of multiple follicular growth factors in human skin cells, including insulin-like growth factor-1, VEGF, and keratinocyte growth factor [117]. Concurrently, a clinical trial involving 22 volunteers using a shampoo containing heat-killed GMNL-653 for 5 months demonstrated reduced dandruff and sebum secretion alongside increased hair growth. The scalp microbiome exhibited elevated abundance of *Mycobacterium globosum* alongside decreased levels of *Mycobacterium restrictum* and *C. acnes* [117]. Scalp microbiome health is closely linked to hair growth, with postbiotics playing a crucial role in regulating scalp microbial balance. These properties position postbiotics as an emerging therapeutic strategy for hair loss conditions like AA. A randomized double-blind controlled study confirmed that a postbiotic cosmetic mimicking platelet-rich plasma growth factor activity markedly improved hair loss severity scores in AA patients, demonstrating markedly superior efficacy compared to placebo [118].

**Fig. 5.**
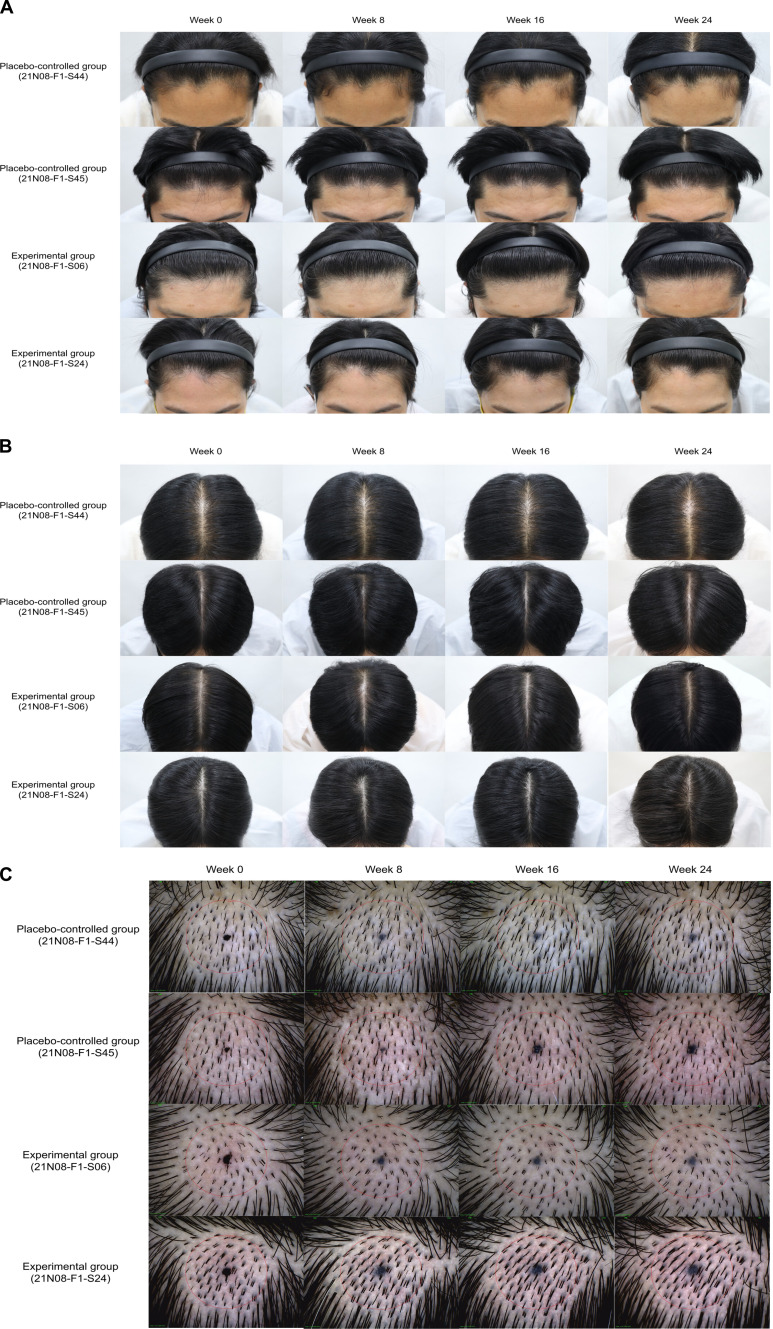
High-resolution photograph of (A) crown area and (B) front hairline. Crown area and front hairline were observed using digital camera. (C) Phototrichogram of placebo-controlled group and experimental group. The phototrichogram images were obtained using a folliscope. Reprinted from [119] under a CC BY 4.0 license.

In the clinical exploration of scalp microbiome modulation for hair loss intervention, topical probiotic formulations represent a key early research direction. Preliminary preclinical studies and a few small-scale human trials have examined the efficacy of topical probiotic applications such as sprays and serums. For instance, Bae et al. conducted clinical trials of heat-treated fermented *Lactobacillus rhamnosus* LM1020 (HT-LM1020) in human follicular dermal papilla cells (HFDPC), scalp tissue, and AGA patients (Fig. 5). They found that HT-LM1020 induces HFDPC proliferation by regulating the cyclin–CDK complex and growth factors such as FGF and EGF. Furthermore, HT-LM-1020 combined with menthol, salicylic acid, and panthenol demonstrated marked improvement in AGA patients. Menthol, salicylic acid, and panthenol are commonly used in hair care products. Menthol alleviates skin discomfort and provides relief by cooling the scalp, salicylic acid is a keratolytic agent that reduces scalp flaking, and panthenol acts as a skin regenerator and moisturizer. These 3 substances do not promote hair growth or treat alopecia. These findings indicate that HT-LM1020 acts as a synergistic factor for anti-hair loss ingredients, promoting cell proliferation and CDK expression [119]. Furthermore, this experimental product has been approved by the Korea Ministry of Food and Drug Safety as a functional cosmetic for anti-hair loss.

In summary, postbiotics promote hair growth through a multitargeted, comprehensive process. These findings reveal the crucial role of microbial metabolites in skin and hair health, providing accumulating preclinical and preliminary mechanistic evidence to support the development of postbiotic-based hair growth products. Microbial strains for hair loss treatment no longer focus solely on inhibiting a single target (such as DHT), but rather aim to restore the scalp’s healthy microbiome, fundamentally eliminating inflammatory and imbalanced factors that cause follicular dysfunction.

Nevertheless, it is necessary to temper the above expectations with current limitations in clinical evidence and translational practice. Most microbiome-based interventions for alopecia remain at the preclinical or early exploratory stage, with few advancing to late-phase clinical evaluation. Available human studies are generally limited by small sample sizes, nonrandomized study designs, and a distinct lack of large-scale, double-blind randomized controlled trials (RCTs), which weakens the strength of efficacy evidence and limits the generalizability of conclusions. Furthermore, for live biotherapeutic products intended for scalp use, there remain substantial regulatory uncertainties in terms of product classification, quality control criteria, and safety evaluation standards across global jurisdictions, posing a key bottleneck for industrial transformation and clinical adoption.

Although currently used primarily as an adjunct or early intervention, its high safety profile and natural mechanism of action make it an attractive component of comprehensive hair loss management strategies. With ongoing research and technological advancements, along with the gradual establishment of high-quality clinical evidence and standardized regulatory systems, microbiome-targeted hair regrowth strategies hold promise for delivering more personalized and fundamental treatment options for hair loss patients in the future.

## Technical Challenges and Future Directions in Utilizing Microbiotas for Hair Regrowth

### Strain stability and consortium design

Live bacteria are highly susceptible to inactivation during preparation, storage, and delivery, which represents a major obstacle to their clinical translation. Strain stability is influenced not only by external environmental conditions, such as temperature and pH, but also by the scalp microenvironment [120]. Therefore, improving the stability of microbial products is a prerequisite for their therapeutic application.

Several strategies may help address this challenge. First, freeze-drying technology has been widely used in the development of oral microbial therapeutics to convert viable strains into lyophilized powders, thereby markedly enhancing storage stability and prolonging shelf life. Research has confirmed that an optimized freeze-drying process can help probiotics maintain high viability during long-term storage. For example, *Saccharomyces boulardii* freeze-dried using a specific blend of cryoprotectants (such as 21.24% lactose, 22.00% trehalose, and 4.00% monosodium glutamate) achieved a survival rate of 64.22% and exhibited a lower inactivation rate constant in accelerated storage tests [121]. Another study showed that freeze-dried *L. plantarum* powder retained a higher and more stable count of live cells than spray-dried samples after being stored at 4 °C for 8 weeks [122]. Second, encapsulation approaches, including microcapsules and nanomaterial-based carriers, can protect strains from unfavorable external conditions and improve their survival during storage and delivery [123]. The air shear-mediated single-bacterial microgel encapsulation technology developed by Wu et al. can markedly enhance the antiinflammatory therapeutic potential of probiotics in inflammatory bowel disease by optimizing their *in vivo* survival and targeted delivery properties [124]. Third, genetic engineering may further enhance strain tolerance and functional activity through targeted gene editing. For example, CRISPR/Cas9 has demonstrated high safety and efficiency in tumor-related gene editing, particularly when combined with nonviral nanodelivery systems. Similar strategies could be applied to modify specific microbial genes, thereby improving strain resilience and viability under harsh conditions [125,126].

In addition to stability, the therapeutic efficacy of a single strain is often insufficient, highlighting the need to explore synergistic interactions among different microorganisms. In this regard, the design of probiotic consortia that better mimic the healthy scalp microbiome may offer a more effective therapeutic strategy. For instance, *L. plantarum* suppresses inflammation, whereas *S. epidermidis* promotes hair follicle cell proliferation and angiogenesis through activation of the PI3K/AKT signaling pathway, thereby accelerating wound healing and stimulating hair follicle regeneration [87]. Combining such strains may therefore improve the scalp microenvironment through complementary mechanisms. Future studies should focus on establishing strain interaction models to identify optimal microbial combinations, while also leveraging synthetic biology to engineer strains with enhanced cooperative effects. Besides, engineered probiotics also offer novel avenues for efficient therapy. Synthetic biology modifications enable strains to sustainably secrete growth factors—such as VEGF-engineered *E. coli* [127]—promising in hair regeneration.

### Targeted transdermal delivery efficiency

Traditional topical formulations, such as shampoos and creams, are often hindered by the scalp’s stratum corneum, which limits microbial strain penetration beyond the epidermis. Because therapeutic efficacy depends on effective delivery to deep hair follicles, several strategies have been developed to overcome this transdermal bottleneck. Nanocarrier technology utilizes nanoparticles to precisely transport microbial strains to target areas, thereby improving penetration and bioavailability [128,129]. Biofilm technology promotes bacterial adhesion and sustained penetration on the skin surface through the use of biocompatible membrane materials [130,131]. In addition, microneedle (MN) patches offer efficient and intelligent solutions for precision drug delivery, while engineered probiotics further enhance targeting capabilities. Among these, MN-based delivery systems have demonstrated particularly strong potential for clinical translation and are discussed in detail in the following sections.

#### Microneedles

As an efficient transdermal delivery method [132], MNs show immense potential for delivering active biological agents, such as probiotics and postbiotics [133–135]. Notably, the regulatory role of MNs in scalp microecology involves 2 conceptually distinct functional dimensions. One is the delivery effect: MNs act as a physical penetration tool to deliver exogenous beneficial microbes/postbiotics to deep hair follicles and dermal tissues, thereby regulating the microecological structure. The other is the physical micro-trauma effect: Empty MNs induce mild controllable trauma and local inflammatory response, whose direct impact on endogenous scalp, follicular, and subcutaneous microecology still lacks direct quantitative evidence. This section focuses predominantly on the delivery effect of MN systems for microbiome-targeted alopecia therapy. Although current studies predominantly focus on wound healing, acne, and atopic dermatitis, their core principles provide a solid theoretical foundation for extrapolating these technologies to scalp microbiome regulation and hair loss treatment [136,137]. Recent transdermal innovations demonstrate how MNs can effectively bypass skin barrier interference to reach deep target tissues. For example, a 2024 study by Zheng et al. developed a microbial MN system powered by *Enterobacter aerogenes*. By adjusting glucose concentrations to regulate gas production, this system actively drives payloads into the skin, achieving delivery depths of 1,000 μm in psoriasis models and pioneering new pathways for deep-tissue drug delivery [138]. Experimental results indicate that MN patches loaded with *S. epidermidis* extracellular vesicles markedly enhance the penetration efficiency of antimicrobial peptides without causing irreversible damage to the skin barrier [139]. Although direct evidence regarding the distribution dynamics of live bacteria and postbiotics within scalp hair follicles, as well as the quantitative impact of specific MN parameters, remains limited, elucidating these factors is crucial for successful clinical translation, as will be systematically detailed below.

##### Spatiotemporal distribution dynamics of MN-delivered postbiotics and live bacteria

MN length is the key parameter determining delivery depth. MNs used for skin treatments typically range from tens to hundreds of micrometers, sufficient to penetrate the epidermis (approximately 50 to 150 μm) and reach the superficial dermis [136] Hair follicle depth varies by anatomical site, and scalp follicles can extend deeply into the subcutaneous tissue. Theoretically, by designing MNs with specific lengths (e.g., 500 to 1,000 μm or longer), probiotics or postbiotics could be delivered directly to the infundibulum, isthmus, or even the peri-dermal papilla region of the hair follicle [137]. However, there are currently no specific data from related studies, marking an area that requires dedicated future research. Studies have shown that MN arrays can successfully deliver substances to the dermis [140], providing a solution for deep tissue bacterial elimination.

The payload release dynamics of MNs depend on their material. Dissolvable MNs realize rapid drug release as the needle body material (e.g., hyaluronic acid and gelatin) dissolves in the skin’s interstitial fluid after puncture [141,142]. For more prolonged effects, sustained release can be achieved using strategies such as core–shell structures [143], nanoparticle encapsulation [144–146], or hydrogel backing layers [147]. For instance, core–shell MNs can initially release antibacterial components, followed by the sustained release of probiotic-derived extracellular vesicles [143]. Alternatively, loading live bacteria (e.g., *Bacillus subtilis*) onto a patch backing allows for long-term colonization on the skin surface after the MNs dissolve [145]. Therefore, postbiotics (e.g., inactivated bacteria and metabolites) may achieve early distribution via rapidly dissolving MNs, whereas live bacteria can maintain a longer-term spatiotemporal presence through sustained-release systems or surface colonization.

##### Impact of MN parameters on microbial survival and colonization efficiency

The fabrication and application parameters of MNs directly affect the viability of the loaded biologicals and the ultimate therapeutic efficacy. When delivering live bacteria, the MN fabrication process (e.g., temperatures during cross-linking and curing, ultraviolet [UV] irradiation, and use of organic solvents) must be mild to ensure microbial viability. Successful cases exist in the literature: MNs fabricated using water-based processing of silk fibroin preserved biosensor activity [148]; bilayered MNs loaded with live *B. subtilis* on the patch backing successfully delivered the bacteria and maintained their viability on the skin surface for over 9 days [145]. Another study directly utilized MN arrays for the intradermal delivery of bacteria, demonstrating their ability to deliver live microbes and trigger an immune response [140]. The Zong team innovatively developed a microbial pneumatic MN system. By integrating the physical delivery advantages of pneumatic MNs with the biological functions of microbial formulations, they achieved precise targeted delivery to the lesion site, effectively enhancing the colonization efficiency and sustained action of microorganisms in the target tissue [149]. These findings indicate that delivering live bacteria via MNs is feasible through appropriate material selection (e.g., biocompatible polymers) and process optimization (e.g., low-temperature molding).

The colonization efficiency of postbiotics or probiotics depends not only on the quantity and viability of the MN-delivered live bacteria but also on the local scalp microenvironment. MN delivery can simultaneously create conditions conducive to probiotic colonization. For example, studies on MNs combined with postbiotics for acne treatment showed that the therapy helps restore the skin barrier and balance the skin microbiota [150]. Similarly, in wound models, MNs delivering probiotic extracellular vesicles were able to increase microbial diversity at the wound site [143]. Thus, the colonization efficiency of MN-loaded postbiotics or probiotics may be synergistically enhanced through the dual action of improving the local microenvironment (e.g., pH, moisture, and nutrients) and directly introducing beneficial bacteria.

##### Interaction between MN-induced micro-trauma inflammatory responses and probiotic immune regulation

MN puncture inevitably causes mild, controllable physical damage, triggering a transient acute inflammatory response that includes cytokine release and immune cell recruitment [140]. This inflammatory response is typically part of the natural healing process. Extensive research demonstrates that probiotics and their derivatives (postbiotics and extracellular vesicles) possess marked immunomodulatory functions. They can suppress pro-inflammatory cytokines, promote the expression of anti-inflammatory cytokines, induce macrophage polarization toward the anti-inflammatory M2 phenotype, and regulate T-cell responses [141,143,151]. In atopic dermatitis models, MN patches containing adipokines and live bacteria were able to restore inflammatory dysregulation [142,144].

### Individual variations in the scalp microbiome and precision intervention

Individual variations in the scalp microbiome may provide an important basis for precision intervention in hair loss disorders. The composition of the scalp microbiome is influenced by multiple factors, including ethnicity, age, geographic environment, and sebum secretion phenotype. For example, studies in Japanese males with AGA have reported increased relative abundance of *Malassezia restricta* and *C. acnes*, accompanied by a decrease in *S. epidermidis* [74,152]. In Korean populations, patients with AGA have been found to exhibit higher α-diversity in the scalp microbiome, which may be associated with the presence of nonresident bacteria [152]. In addition, scalp microbiome composition appears to vary across ethnic and geographic backgrounds; for instance, the relative abundance of *C. acnes* and *Malassezia* has been reported to differ between Asian and European populations [152,153].

These observations suggest that scalp microbiome-targeted strategies may not be equally applicable across all individuals. Instead, host characteristics, disease subtype, and baseline microbial features should be taken into account when evaluating microbiome-associated interventions. In this context, establishing population-specific reference intervals for the scalp microbiome may help improve both disease stratification and the development of more individualized management approaches (Table 6).

**Table 6.** Population-specific reference interval construction strategies for the scalp microbiome

Source of heterogeneity	Strategy for constructing population-specific reference intervals	Methods	Ref.
Ethnic differences	Construct baseline values and abundance thresholds for core scalp microbiota separately for different ethnic groups (e.g., Caucasian, Asian, and African populations).	16S rRNA/metagenomic sequencing, population difference analysis, genetic background correction	[85,189]
Age differences	Since sebum secretion and microbial dynamics differ among children, adolescents, adults, and older adults, establish age-dependent reference ranges for microbial abundance.	α/β diversity analysis, core microbiota screening, age regression fitting	[65]
Geography and climate	Recruit study populations by climatic zone (temperate, tropical, and frigid) and humidity type (humid and arid) to ensure regional and climatic representativeness of the cohort; standardize sampling seasons and construct region-specific microbial reference intervals.	Environmental factor association analysis (RDA/CCA), regional microbiota difference identification	[173]
Scalp sebum secretion phenotype	Classify phenotypes according to low, medium, and high sebum secretion levels, combined with hair loss grading, to establish phenotype-matched normal ranges of microbiota.	Scalp sebum quantification, microbiome phenotype association analysis	[85]

Such a framework would require large-scale, multicenter cohort studies involving participants from different ethnic groups, age ranges, and geographic regions. By comparing healthy controls with disease populations, these studies may help define baseline microbial characteristics and identify thresholds associated with dysbiosis, as illustrated in Japanese and Korean AGA cohorts [152]. For real-world clinical implementation, population-specific reference ranges should be further stratified by sebum secretion phenotype, age stratum, and ethnic background, rather than adopting a universal normal threshold. In routine clinical testing, each subject’s microbiome profile can be automatically matched to the corresponding reference interval of their population subgroup, enabling more accurate grading of dysbiosis and reducing misclassification caused by interpopulation baseline differences. To improve comparability across studies, standardized sampling methods, such as scalp swabs, should be combined with high-throughput sequencing approaches, including 16S rRNA and internal transcribed spacer (ITS) sequencing. To translate these protocols into daily dermatological practice, consensus standard operating procedures (SOPs) need to be established, unifying key parameters including sampling anatomical sites (e.g., vertex lesional area and matched occipital control area), presampling scalp cleansing rules (e.g., no shampooing for 48 h before sampling), swab material type, and standardized wiping force and stroke counts. Harmonized SOPs will ensure consistent and comparable microbiome test results across different clinical centers, laying an operational foundation for widespread clinical application. The resulting datasets could then be analyzed using machine learning methods to identify microbial features associated with hair loss, such as changes in the abundance of *C. acnes* and *Malassezia* [153,154].

One example of this translational direction is the Scalp Microbiome Health Index (MiSCH), developed by the Bioinformatics Research Group at Qingdao University’s School of Computer Science and Technology in collaboration with the Departments of Pathology and Dermatology of its affiliated hospital. According to the reported study, MiSCH was designed to quantify AGA severity and identify individuals at increased risk, and its integration with artificial intelligence (AI) may provide support for more individualized clinical assessment [154]. In clinical practice, such quantitative indices can be embedded into hospital information systems to automatically generate dysbiosis scores and risk stratification reports, lowering the threshold for clinicians to interpret microbiome results and facilitating point-of-care decision-making. However, the clinical utility of such tools still requires further validation in independent and diverse populations.

At the disease level, specific microbial patterns may also provide clues for tailored intervention. For example, increased abundance of *Malassezia furfur* has been reported in the hair follicle microbiome of patients with AA, suggesting a possible association between fungal dysbiosis and disease status. On this basis, modulation of the local microbial community, including approaches aimed at restoring bacterial–fungal balance, may represent a potential therapeutic direction. Nevertheless, the efficacy and safety of such targeted microbiome-based interventions remain to be established by further mechanistic and clinical studies.

In the future, portable detection platforms may facilitate a more integrated workflow of “detection–analysis–intervention”, although such applications are still largely conceptual at present. In addition, functional prediction tools, including Kyoto Encyclopedia of Genes and Genomes pathway analysis, may help reveal metabolic differences associated with distinct microbial patterns and thereby refine patient stratification [155]. These efforts should also be complemented by longitudinal studies to monitor microbiome dynamics over time and to evaluate how microbial alterations change in response to treatment. For routine clinical management, a standardized longitudinal monitoring workflow can be established with fixed follow-up time points: baseline testing before intervention initiation, and retesting at 1, 3, and 6 months after treatment. Longitudinal dynamic tracking captures individual trajectories of microbiome recovery, which is more predictive of therapeutic response than a single cross-sectional test. In addition, each patient can serve as their own control by establishing a personal baseline microbiome profile, allowing intervention strategies to be adjusted in real time according to individual dynamic changes, rather than relying solely on population-level reference values. This personalized monitoring mode better aligns with the core principles of precision medicine for alopecia management. For example, *Limonia strychnifolia* root extract and heat-treated *Limosilactobacillus fermentum* LM1020 have been reported to improve microbial balance and promote hair growth in experimental or preliminary studies [119,153]. Taken together, current evidence supports the potential relevance of individual scalp microbiome variation in future precision management, while its translation into routine personalized therapy still requires more robust clinical evidence.

### Industrial scale-up, quality control, and regulatory strategy

Achieving clinical translation of microbial hair loss therapies requires overcoming interconnected challenges in large-scale production, quality assurance, and regulatory navigation. Rather than treating these as abstract barriers, this section outlines each bottleneck alongside concrete, industry-grounded technical and strategic solutions.

#### Large-scale production and process engineering

Under laboratory conditions, microbial strain cultivation and optimization typically occur in small-scale, tightly controlled environments. However, scaling up to industrial production introduces environmental stress, nutritional limitations, and competitive microbial influences that can cause functional decline or genetic drift. Ensuring strain stability, metabolic activity, and production efficiency at scale therefore constitutes the primary engineering challenge.

Three specific process improvements, already validated in the oral probiotic industry, merit priority investigation for microbial hair loss therapies. First, transitioning from conventional batch fermentation to fed-batch or continuous fermentation with real-time process analytical technology (PAT)—including dissolved oxygen, pH, and metabolite monitoring via Raman spectroscopy or near-infrared probes—can dramatically improve yield and consistency. In the probiotic sector, fed-batch cultivation of *Lactobacillus salivarius* I 24 achieved an 8-fold increase in viable cell concentration (10.7 × 10^10^ CFU/ml) and a 26-fold increase in cell yield per unit glucose compared to batch cultivation [156]. Similarly, constant fed-batch fermentation of *Pediococcus acidilactici* yielded a maximum viable cell concentration 6.1 times higher than batch mode, with a concurrent 1.6-fold reduction in inhibitory lactic acid accumulation [157]. For *Lactobacillus casei* grown in whey-based medium, fed-batch cultures achieved a volumetric productivity of 0.45 g l^−1^ h^−1^, approximately 4-fold higher than both batch and continuous cultures (0.11 g l^−1^ h^−1^) [158]. These benchmarks demonstrate that advanced fermentation strategies can deliver multifold improvements in viable cell output while reducing batch-to-batch variation, a critical prerequisite for pharmaceutical-grade production.

Second, for postbiotic production, spray-drying with protective matrices offers a scalable and cost-effective alternative to freeze-drying. As discussed in the “Strain stability and consortium design” section, drying method selection is strain-dependent; at industrial scale, the key additional consideration is energy cost and throughput. Freeze-drying requires up to 3 times the energy consumption of spray-drying [159,160], making it an important cost barrier at industrial scale. Meanwhile, optimized spray-drying protocols have demonstrated high cell viability retention: *L. plantarum* BG24 spray-dried with reconstituted skim milk and yeast extract at optimized inlet/outlet temperatures (150 °C/83 °C) achieved 92.23% viability, with moisture content and water activity suitable for long-term storage [161]. Protective carbohydrate matrices—including maltodextrin, trehalose, and their blends—further enhance survival by stabilizing cell membranes during the dehydration process [159,162]. However, it should be noted that the optimal drying method and protective formulation are strain-dependent, and spray-drying can cause greater viability loss than freeze-drying for some heat-sensitive strains [163]. Therefore, strain-specific optimization is essential.

Third, for topical formulations containing live organisms, microencapsulation using alginate–chitosan coating represents a promising approach for protecting viable bacteria in the acidic scalp surface environment (pH 4.5 to 5.5). In the food and oral pharmaceutical sectors, chitosan-coated alginate microcapsules have been extensively validated for protecting probiotics under acidic conditions: the polyelectrolyte complex formed between negatively charged alginate and positively charged chitosan creates a semipermeable membrane with reduced permeability to water-soluble molecules, effectively shielding encapsulated bacteria in simulated gastric fluid (pH 1.5 to 3.0) [164,165]. Alginate–chitosan double-layer encapsulation of lactic acid bacteria via extrusion, emulsion, or spray-drying methods maintained probiotic viability for at least 6 months at room temperature [166]. Furthermore, chitosan-coated microcapsules enable pH-responsive controlled release, with bacteria released preferentially at near-neutral pH rather than in acidic environments [167]. While these studies have focused on gastrointestinal delivery, the underlying acid-protection and controlled-release principles are directly applicable to the scalp microenvironment, and validation of this approach for dermal/follicular delivery should be a priority for future research.

#### Quality control framework

As biological agents, microbial strains demand quality control standards far more stringent than those for conventional small-molecule pharmaceuticals. Rigorous oversight of purity, potency, safety, and batch consistency is essential. During cultivation, strains may produce toxins or elicit unintended immune responses, necessitating detection through advanced analytical techniques. Concurrently, storage, transportation, and usage conditions must be standardized to guarantee efficacy and safety in clinical applications.

To address these requirements, the field should adopt a tiered analytical framework comprising 3 levels: (a) Identity verification: Whole-genome sequencing of each production lot to confirm strain identity and detect contaminating organisms or horizontal gene transfer events—moving beyond simple phenotypic characterization. (b) Potency assays: Standardized functional in vitro readouts tailored to the strain’s mechanism of action—such as NF-κB inhibition assays for anti-inflammatory strains or agar diffusion antimicrobial activity assays for pathogen-excluding strains—rather than reliance on simple CFU counts, which do not reflect biological function. (c) Safety profiling: A validated panel including endotoxin quantification (limulus amebocyte lysate assay), antibiotic resistance gene screening, and cytotoxicity assessment on human keratinocyte cell lines to establish a comprehensive safety dossier for each candidate strain.

The 2025 Preferred Reporting Items for Microbiological Therapies (PRIM 2024) guidelines, developed by Zhang et al. [168] through an international Delphi consensus, provide a valuable cross-disciplinary reporting framework that enhances comparability and scientific rigor across microbiological therapy research. Additionally, AI-based hair counting algorithms and ultrahigh-resolution confocal microscopy have been recommended for standardized verification of hair growth efficacy, though these measurement standards have yet to gain widespread adoption. Nonetheless, PRIM 2024 remains a reporting guideline; the field urgently needs product-specific pharmacopeial monographs analogous to those established for injectable biologics to serve as binding quality standards.

#### Regulatory pathways and market access strategy

As an emerging therapeutic modality, the regulatory pathway for microbial strains remains incompletely defined. Heterogeneity among national drug regulatory authorities in approval criteria, clinical trial design requirements, and safety assessment standards creates uncertainty and inflates R&D costs. Furthermore, long-term safety and potential side effects require validation through large-scale clinical trials, further prolonging development timelines.

For live microbial products intended as prescription treatments, the Food and Drug Administration’s (FDA’s) 2016 Early Clinical Trials with Live Biotherapeutic Products guidance and the European Medicines Agency’s (EMA’s) 2019 Reflection Paper on LBPs provide established regulatory frameworks. These require Phase I–III clinical trials with well-defined endpoints, including scalp microbiome modulation (assessed via 16S rRNA sequencing), validated hair density measurements (phototrichogram), and patient-reported outcome measures (e.g., the Hair Specific Skindex-29). Manufacturers are strongly encouraged to engage regulatory agencies through presubmission meetings (pre-IND meetings with FDA; Scientific Advice with EMA) to reach agreement on primary endpoints and trial design before committing to costly Phase III programs.

For postbiotic products with well-characterized compositions (inactivated bacteria and purified metabolites), manufacturers should pursue the functional cosmetic or quasi-drug regulatory pathway available in several jurisdictions. Specifically, the Korea MFDS functional cosmetics pathway, the Japan PMDA quasi-drug framework, and the EU Cosmetic Regulation (EC 1223/2009) offer faster market access with lower clinical evidence thresholds relative to pharmaceutical registration. The successful approval of HT-LM1020 as a functional cosmetic by the Korea MFDS provides a concrete and replicable precedent for this approach.

The financial burden of clinical development for microbial therapies—particularly the large-scale RCTs required for LBP approval—represents an important barrier, especially for small and mid-sized biotech companies. Public–private partnership models offer a viable path forward: frameworks such as the Innovative Medicines Initiative in Europe, or collaborative agreements with academic medical centers, can distribute the costs of clinical trials while maintaining independent oversight of efficacy and safety data. Such models have successfully accelerated translation in analogous fields, including fecal microbiota transplantation for *Clostridioides difficile* infection.

**Fig. 6.**
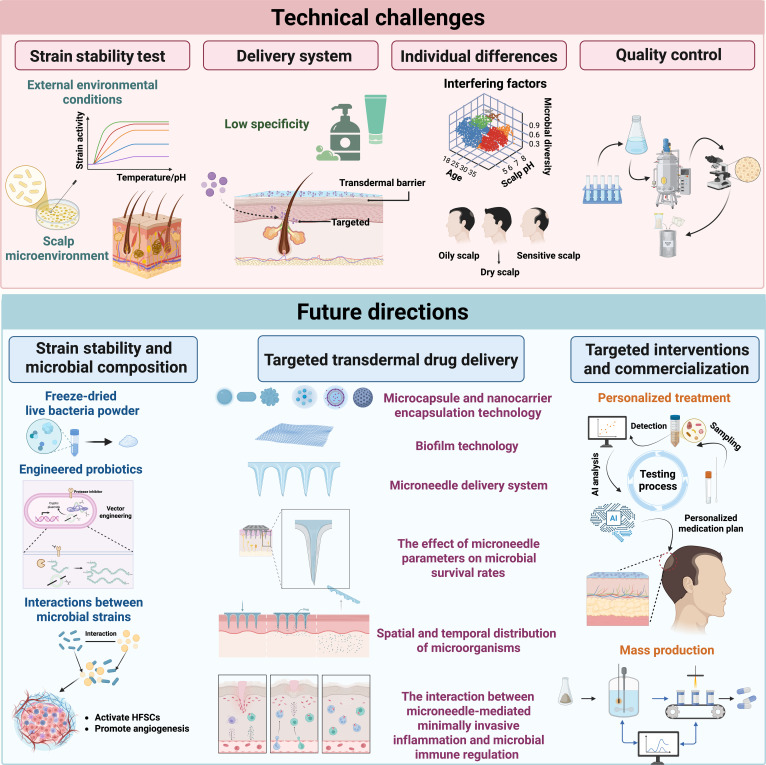
Technical challenges and future directions in utilizing microbiotas for hair regrowth.

In summary, the clinical translation of microbial hair loss therapies must overcome tightly coupled challenges in strain stability, delivery efficiency, production scalability, and regulatory clarity. The solutions outlined above—continuous fermentation with PAT, tiered quality control analytics, dual-track regulatory navigation, and collaborative funding models—provide an actionable roadmap grounded in existing industrial precedent and regulatory infrastructure. Advancing this roadmap will require sustained interdisciplinary collaboration among microbiologists, bioprocess engineers, regulatory scientists, and dermatologists to move microbial therapies from the laboratory to clinical practice, ultimately delivering more effective treatment options for individuals experiencing hair loss (Fig. 6).

## Summary and Outlook

Hair loss represents a substantial global health burden because of its high prevalence and its adverse effects on psychological well-being and quality of life. Although conventional treatments, including minoxidil, finasteride, JAK inhibitors, and hair transplantation, can provide clinical benefit, their effectiveness varies considerably among patients, and durable disease control remains difficult in many alopecia subtypes. The evidence synthesized in this review supports the scalp microbiome as an important component of the broader biological network regulating scalp barrier integrity, immune homeostasis, sebum metabolism, follicular regeneration, and hair cycling.

This review advances a conceptual framework for understanding the scalp microbiome–hair axis at both disease and mechanistic levels. Cross-subtype comparison suggests a 2-layer model in which different alopecia forms share a conserved pathological layer—including barrier disruption, innate immune activation, altered lipid or metabolite processing, and impaired anagen support—while retaining subtype-selective mechanisms. These include immune privilege instability in AA, androgen–lipid–microbiome coupling in AGA, *Malassezia*-centered inflammatory ecology in seborrheic dermatitis-associated hair loss, and infection–biofilm–neutrophilic injury progressing toward stem-cell depletion and fibrosis in scarring alopecia. In parallel, the proposed 3-tier pathogenic cascade links upstream microbial perturbations and host-sensing events to midstream signaling hubs, including NF-κB, JAK–STAT, PI3K/AKT, and Wnt/β-catenin, and ultimately to specific follicular outcomes such as stem-cell quiescence, dermal papilla dysfunction, vascular regression, immune privilege collapse, and fibrosis. These frameworks help explain why microbiome-targeted interventions are unlikely to be universally effective and why future therapeutic strategies will require stratification by both clinical phenotype and microbiome state.

Despite this conceptual and translational progress, the current evidence base has important limitations. Most human studies are small, cross-sectional, and observational, while mechanistic conclusions often rely on preclinical experiments conducted in nonscalp skin, isolated cell systems, or animal models. Considerable heterogeneity in presampling scalp cleansing, anatomical sampling sites, specimen-collection methods, sequencing platforms, reference databases, bioinformatic pipelines, and patient-selection criteria further limits reproducibility and cross-study comparability. In addition, ethnic background, geographic environment, age, sex, sebum production, scalp-care practices, medication use, and disease severity are not consistently controlled. Consequently, many reported microbial signatures remain population- or protocol-dependent and cannot yet be regarded as clinically validated biomarkers.

Establishing causality remains another major scientific challenge. It is not yet clear whether specific microbial alterations initiate follicular dysfunction, amplify established disease, or arise secondarily from changes in sebum composition, inflammation, androgen signaling, immune activity, or follicular architecture. The scalp microbiome also interacts dynamically with host endocrine rhythms, barrier function, local immunity, environmental exposures, and treatment history. These bidirectional relationships make it difficult to distinguish primary microbial drivers from secondary ecological consequences. Moreover, the relative contribution of the microbiome is likely to differ among alopecia subtypes and disease stages. Microbial dysbiosis may be an important amplifier in nonscarring alopecia but may become particularly consequential in inflammatory and scarring disorders, where persistent infection, biofilm formation, and immune activation can contribute to irreversible follicular destruction.

Clinical translation is further constrained by challenges in strain selection, formulation stability, targeted follicular delivery, manufacturing scalability, quality control, and regulation. Live microorganisms may lose viability during production, storage, or topical application, whereas postbiotics offer greater stability but require validated potency assays that reflect biological activity rather than product concentration alone. Effective delivery to the follicular infundibulum and deeper follicular compartments also remains difficult. Although microencapsulation, nanoformulations, and MN-assisted delivery may improve localization and retention, their safety, dosing, and effects on microbial ecology require systematic evaluation. Regulatory classification is similarly complex because live biotherapeutics, postbiotics, cosmetics, quasi-drugs, and combination products may be subject to different manufacturing and clinical-evidence requirements across jurisdictions.

These limitations define several actionable priorities for future research. First, standardized multicenter cohort studies should adopt harmonized protocols for scalp cleansing, anatomical site selection, sample collection, storage, sequencing-library preparation, reference-database selection, and bioinformatic analysis. Cohorts should include participants from diverse ethnic, geographic, age, sex, and disease-severity groups and should systematically record relevant covariates, including sebum production, scalp-care practices, medication exposure, diet, and environmental factors. Such standardization would improve reproducibility, enable meaningful cross-population comparisons, and support the establishment of population-specific microbiome reference intervals and clinically relevant dysbiosis thresholds.

Second, future studies should integrate metagenomics with metabolomics, host transcriptomics, lipidomics, proteomics, and spatial profiling. Taxonomic abundance alone cannot determine whether a microorganism is functionally active or whether its metabolites directly affect follicular biology. Multi-omics integration should therefore be used to identify microbial metabolic products, host receptors, signaling pathways, immune states, and lipid alterations that distinguish active pathogenic processes from passive colonization. Spatially resolved analysis of the scalp surface, follicular infundibulum, sebaceous gland, bulge region, and perifollicular tissue will be particularly important because microbial composition and host responses vary across follicular compartments.

Third, causal microbiome-manipulation studies should test temporal association, biological sufficiency, mechanistic necessity, and therapeutic reversibility. Longitudinal human cohorts should determine whether microbial alterations precede disease progression or treatment response. Ex vivo human hair follicles, reconstructed scalp models, and follicular organoids can be exposed to defined strains, microbial consortia, or metabolites to assess their direct effects on barrier function, inflammatory signaling, dermal papilla activity, stem-cell behavior, and hair-shaft elongation. Germ-free or antibiotic-conditioned animal models reconstituted with human scalp microbiota, together with targeted monocolonization or defined-community transplantation, may clarify whether candidate microorganisms are sufficient to induce relevant phenotypes. Pathway-inhibition and rescue experiments should further determine whether TLR, AhR, NF-κB, JAK–STAT, PI3K/AKT, Wnt/β-catenin, or TGF-β/BMP signaling is necessary for microbiome-associated follicular effects.

Fourth, candidate microbial biomarkers should progress through a staged validation pipeline. Initial discovery should be followed by analytical validation in independent multicenter cohorts and prospective clinical validation in predefined patient populations. Biomarker panels should be evaluated for diagnostic accuracy, prognostic value, treatment-response prediction, reproducibility, and incremental benefit over existing clinical measures. Wherever possible, microbial signatures should be combined with host inflammatory markers, lipid profiles, clinical phenotype, and treatment history rather than being interpreted in isolation. This approach may support more robust patient stratification than any single taxon or diversity metric.

Fifth, microbiome-based therapies should be assessed in rigorously designed clinical trials using standardized formulations, clearly defined microbial and clinical endpoints, and adequate follow-up. Head-to-head studies should compare microbiome-directed interventions with placebo, conventional therapy, and combination regimens involving treatments such as minoxidil, finasteride, antifungal agents, or JAK inhibitors. Longitudinal monitoring should determine whether clinical improvement is accompanied by sustained microbial remodeling, normalization of host molecular pathways, and durable benefit after treatment discontinuation. Trials should also identify which patients are most likely to benefit according to alopecia subtype, baseline microbiome state, inflammatory phenotype, and disease stage.

Finally, translation into routine practice will require rapid, affordable, and clinically interpretable detection technologies. Portable sampling and microbiome-analysis platforms, coupled with validated computational or AI-assisted models, may eventually support an integrated “detection–analysis–intervention” workflow. However, such systems must undergo transparent training, external validation, bias assessment, and prospective clinical evaluation before they can be used for treatment selection or response monitoring.

In conclusion, microbiome modulation represents a promising but still emerging therapeutic strategy for alopecia. Current evidence supports the biological plausibility of targeting microbial ecology, microbial products, and host–microbe signaling networks, but does not yet justify considering the scalp microbiome an independently established causal driver across all hair-loss disorders. Progress will depend on standardized study design, functional multi-omics, rigorous causal experimentation, validated biomarker pipelines, and well-controlled clinical trials. Through coordinated collaboration among microbiologists, dermatologists, systems biologists, bioengineers, clinical trialists, and regulatory scientists, the scalp microbiome–hair axis may ultimately provide a foundation for more precise, mechanistically informed, and durable approaches to the prevention and treatment of hair loss.

## Ethical Approval

No animals or humans were involved in this study.
